# Tuft cells utilize taste signaling molecules to respond to the pathobiont microbe *Ruminococcus gnavus* in the proximal colon

**DOI:** 10.3389/fimmu.2023.1259521

**Published:** 2023-10-25

**Authors:** Hao Lei, Defu Yu, Yan-Bo Xue, Yi-Hong Li, Shi-Meng Gong, Yuan-Yuan Peng, Kai-Fang Liu, Damiano Buratto, Yisen Yang, Sai-Sai Zhang, Min Wu, Ruhong Zhou, Liquan Huang

**Affiliations:** ^1^ College of Life Sciences, Zhejiang University, Hangzhou, Zhejiang, China; ^2^ Zhejiang University Shanghai Institute for Advanced Study, Shanghai, China; ^3^ Monell Chemical Senses Center, Philadelphia, PA, United States

**Keywords:** proximal colon, tuft cells, gut microbes, gasdermin, Tas2Rs, signal transduction, G γ13, *Trpm5*

## Abstract

Tuft cells are a type of rare epithelial cells that have been recently found to utilize taste signal transduction pathways to detect and respond to various noxious stimuli and pathogens, including allergens, bacteria, protists and parasitic helminths. It is, however, not fully understood how many different types of pathogens they can sense or what exact molecular mechanisms they employ to initiate targeted responses. In this study, we found that an anaerobic pathobiont microbe, *Ruminococcus gnavus* (*R. gnavus*), can induce tuft cell proliferation in the proximal colon whereas the microbe’s lysate can stimulate these proximal colonic tuft cells to release interleukin-25 (IL-25). Nullification of the *Gng13* and *Trpm5* genes that encode the G protein subunit Gγ13 and transient receptor potential ion channel Trpm5, respectively, or application of the Tas2r inhibitor allyl isothiocyanate (AITC), G protein Gβγ subunit inhibitor Gallein or the phospholipase Cβ2 (PLCβ2) inhibitor U73122 reduces *R. gnavus*-elicited tuft cell proliferation or IL-25 release or both. Furthermore, *Gng13* conditional knockout or *Trpm5* knockout diminishes the expression of gasdermins C2, C3 and C4, and concomitantly increases the activated forms of caspases 3, 8 and 9 as well as the number of TUNEL-positive apoptotic cells in the proximal colon. Together, our data suggest that taste signal transduction pathways are not only involved in the detection of *R. gnavus* infection, but also contribute to helping maintain gasdermin expression and prevent apoptotic cell death in the proximal colon, and these findings provide another strategy to combat *R. gnavus* infection and sheds light on new roles of taste signaling proteins along with gasdermins in protecting the integrity of the proximal colonic epithelium.

## 1 Introduction

Gut microbiota contains many species of microbes, including viruses, phages, bacteria, archaea, protozoans and fungi. Their interactions with the host cells can be beneficial or harmful to the host, by helping maintain normal physiology of the host or contributing to the pathogenesis of many diseases (1). The cellular and molecular mechanisms underlying the pivotal reciprocal interactions between host cells and many gut microbes are yet to be fully understood.

*Ruminococcus gnavus* (*R. gnavus*) is an obligate anaerobic, Gram-positive commensal firmicutes bacterium that resides in the large intestine of 90% of adults and usually represents less than 0.1% of the gut microbiota (2). It has been found to be associated with many disorders including allergy, asthma, and diarrhea (3, 4) and its elevated abundance of up to 69% has been found in some patients with Crohn’s disease, inflammatory bowel disease, pouchitis, diverticulitis as well as COVID-19 (5–11). *R. gnavus* is noted to be capable of colonizing the crypts of the proximal colon and degrading the mucus layer covering the colonic epithelium, conferring to the host cells the accessibility to the microbes, triggering host immune responses (8, 12). *R. gnavus* may activate intestinal epithelial cells to release cytokines, triggering type 2 innate immune responses via type 2 innate lymphoid cells (ILC2s), leading to an asthmatic inflammatory cascade (3). However, *R. gnavus* can exert multiple effects on the gut epithelial cells. Its metabolites such as phenethylamine and tryptamine can activate trace amine-associated receptor 1 (TAAR1) on the enterochromaffin cells, stimulating the biosynthesis of serotonin, leading to the diarrhea-predominant irritable bowel syndrome (4) while other metabolites, e.g., glucorhamnan polysaccharides, can regulate the tumor necrosis factor TNFα secretion, modulating inflammatory responses (13, 14). The exact mechanisms underlying the host cell-pathogen interactions are not fully understood.

The dynamic changes in the gut luminal contents are indeed constantly monitored by the intestinal epithelial cells, including a rare type of chemosensory cells, tuft cells (15). These cells have so far been found not only in the intestines and stomach, but also in the nasal cavity, trachea, gall bladder, and thymus (16–20). These cells are able to detect various irritants and pathogens including allergens, bacteria, protists and parasitic helminths by taking advantage of different sets of receptors such as the succinate receptor (Sucnr1), free fatty acid receptor 2 (FFAR2), bitter taste receptor (Tas2rs), vomeronasal receptor (Vmn2r26) (21–32). Activation of these receptors in turn stimulates intracellular signaling cascades, which eventually leading to the output effector molecules that are transmitted onto adjacent epithelial cells, immune cells or nerve fibers (33). Canonical taste signaling proteins, including the G protein α and γ subunits Gα-gustducin and Gγ13, the phospholipase Cβ2 (PLCβ2) and transient receptor potential ion channel Trpm5, are highly expressed in tuft cells, making up core signal transduction pathways (15, 16, 22, 25, 32, 34). Activation of these pathways engenders the release of a variety of output signaling molecules, including interleukin-25 (IL-25), acetylcholine, cysteinyl leukotrienes and prostaglandins, resulting in neuronal and immune responses (27, 28, 32, 35). It is, however, not known how intestinal tuft cells may respond to *R. gnavus*.

Based on gene expression profiles, tuft cells can be classified into two subtypes (36), each of which seems to have its own sensing targets and regulatory mechanisms for their responses (28). It is not fully understood how diverse the pathogenic stimuli and output effector molecules are that can be detected and released, respectively, by tuft cells, and what intracellular signal transduction pathways tuft cells employ to convert particular input signals into their respective output effectors to act on downstream cells. In this study, we investigated the responses of mouse proximal colonic tuft cells to the infection of an anerobic bacterium, *R. gnavus*, and found that the Gγ13-Trpm5 signaling circuit is critical to *R. gnavus*-evoked IL-25 release as well as concomitant tuft cell proliferation. Furthermore, this signaling circuit is also essential for maintaining Gasdermin expression and protecting the proximal colonic cells from apoptotic cell death.

## 2 Materials and methods

### 2.1 Animals

C57/BL6 mice were purchased from the Shanghai SLAC Laboratory Animal Company (Shanghai, China). Trpm5-lacZ, ChAT-Ires-Cre and Rosa26-tdTomato (also known as Ai9) mouse lines (Jax stock numbers 005848, 006410 and 007909, respectively) were obtained from the Jackson Laboratory (22, 37, 38). In the Trpm5-lacZ mice, part of the Trpm5-coding region was replaced by the lacZ sequence to express β-galactosidase. ChAT-Ires-Cre mice carrying the internal ribosome entry site and Cre recombinase gene after the choline acetyltransferase (ChAT) coding sequence were crossed with Ai9 to generate ChAT-Ai9 mice in which ChAT-expressing tuft cells are labeled by tdTomato. The Gng13^flox/flox^ mice were generated previously (39), which were bred with ChAT-Ires-Cre mice to generate conditional Gng13 knockout mice (Gng13-cKO), in which the expression of the Gng13-encoded G protein subunit Gγ13 is abolished in the ChAT-expressing intestinal tuft cells. All mice were bred and maintained in the Laboratory Animal Center of Zhejiang University. All experiments with animals were conducted in accordance with the National Institutes of Health (NIH) guidelines for the care and use of laboratory animals and approved by the Animal Care and Use Committee of Zhejiang University.

### 2.2 Preparation of the anaerobic bacterium *Ruminococcus gnavus*, parasitic helminth *Trichinella spiralis* and their lysates

*Ruminococcus gnavus* (*R.gnav*) (ATCC 29149) was obtained from American Type Culture Collection and cultured in ATCC 158 RGCA medium under anaerobic conditions (80% N_2_, 10% H_2_, 10% CO_2_) at 37°C in an anaerobic chamber for ~18 hours. Bacterial growth curves were determined by counting the number of bacteria at given time points using Hungate’s roll-tube method (40). Cells at the end of the exponential growth phase were collected by three consecutive centrifugations at 4000x g for 40 min: after the first centrifugation, the pellet was resuspended in 1x oxygen-free PBS while the supernatant was subject to a second centrifugation, which was repeated once more. Part of the resuspended *R. gnavus* cells was used to inoculate mice by oral gavage or to prepare its lysate via ultrasonic crushing. The protein concentration of the lysate was determined using BCA assay and adjusted to 1.3 mg/ml, which was further diluted to a series of working concentrations in the subsequent experiments.

*Trichinella spiralis* (*T. spiralis*) strain (isolate code ISS634) was maintained in rodents and collected as previously described (22). About 5,000 muscle larvae in 0.5 ml 1xPBS were ultrasonically treated to prepare the *T. spiralis* lysate with a protein concentration of ~2.4 mg/ml, which was diluted to 15% (volume/volume) as a working concentration used in the subsequent assays.

### 2.3 Administration of *Ruminococcus gnavus* and bitter tasting compounds to mice

*R. gnavus* bacteria were administered orally to mice. To increase the colonization efficiency of the bacteria, 8-12 weeks-old mice were deprived of food and water for 6 hours before being orally gavaged with 3 x 10^7^ CFUs of *R. gnavus*. Seven days post the first infection, the mice were administered again with the same amount of *R. gnavus*. At 21 days post the 1^st^ infection, colonization of *R. gnavus* was confirmed by qPCR with the primers for the 16S rRNA genes on the DNA samples extracted from fecal samples. The successfully infected mice were used for experiments.

To stimulate colonic epithelial cells, a mixture of 0.1 ml bitter tasting compounds containing 5 mM salicin and 5 mM quinine was anally administered twice a day for 7 days while control mice were administered with an equal volume of 1xPBS. The mice were sacrificed on day 8 for intestinal tissue analysis.

### 2.4 Immunohistochemistry

The processing and immunostaining of intestinal tissue sections were performed as previously described (22). Briefly, the intestines of *R. gnavus*-infected and uninfected control mice were cut into six segments: duodenum, jejunum, ileum, and proximal, mid and distal colons, and fixed on ice in 4% paraformaldehyde (PFA) for 3 h. The tissues were cryoprotected in 30% (w/v) sucrose overnight and embedded in the O.C.T. Compound embedding medium (Tissue-Tek) before being sliced into 14 μm-thick sections on a cryostat (Thermo scientific, HM525). The sections were then washed in 1x PBS and blocked in the blocking buffer (3% BSA, 0.3% Triton X-100, 2% donkey serum and 0.1% sodium azide in 1x PBS) for 2 h at room temperature. All primary antibodies used in this study were incubated at 4°C overnight and the secondary antibodies were applied at room temperature for 1 h. Primary antibodies used were as follows: anti-Dclk1 (Abcam, ab31704), Alexa Fluor^®^ 488 Rabbit monoclonal to DCAMKL1 (Abcam,ab202754), anti-PLCß2 (Santa Cruz Biotechnology, sc-206), anti-GSDMC2-GSDMC3 (Abcam, ab229896), anti-CD45 (Abcam, ab10558), anti-Muc2 (Abcam, ab272692), anti-cleaved caspase3 (Cell Signaling Technology, #9664), anti-cleaved caspase8 (Cell Signaling Technology, #8592), and anti-cleaved caspase9 (Cell Signaling Technology, #9509). The specificities of these primary antibodies were validated in the previous studies, respectively (22, 27, 41–47). Secondary antibodies used were as follows: goat anti-rabbit IgG H&L (Alexa Fluor^®^ 488) (Abcam. ab150077), goat anti-rabbit IgG H&L (Alexa Fluor^®^ 568) (Abcam, ab175471). DAPI was used for nuclear staining. Fluorescent images were captured using an Olympus FV3000 confocal laser-scanning microscope.

For some tissue sections from the Trpm5-lacZ mice, X-gal staining was used to colocalize Trpm5 with another protein to tuft cells (22). After being labeled with a primary antibody followed by a secondary antibody, sections were washed once in the permeabilization buffer (2 mM MgCl_2_, 0.02% NP40 in 1x PBS) and twice in the washing buffer (2 mM MgCl_2_ in 1x PBS) for 5 min at room temperature and then rinsed in the detergent solution (2 mM MgCl_2_, 0.02% NP40, 0.01% deoxycholate in 1x PBS) for 10 min. The sections were incubated in the β-galactosidase substrate (2 mM MgCl_2_, 0.02% NP40, 0.01% deoxycholate, 5 mM K_3_Fe (CN)_6_, 5 mM K_4_[Fe (CN)_6_]·3H_2_O, 1mg/ml X-gal in 1x PBS) in the dark for 5 h at room temperature. Fluorescent and bright-field images were acquired with a fluorescence microscope (Nikon AZ100).

### 2.5 TUNEL staining

To detect apoptosis in tissues, we employed TUNEL staining to visualize nuclear DNA fragmentation in the nuclei (48). The intestinal tissue sections were prepared in the same way as described above for immunohistochemistry. The sections were washed twice for 10 min with 1xPBS, and then washed once for 5 min with PBS-T containing 0.5% Triton X-100 at room temperature. One Step TUNEL Apoptosis Assay Kit (Beyotime Biotechnology C1086) was used for TUNEL staining according to the manufacturer’s instructions.

### 2.6 Quantitative reverse transcription-PCR analysis on the colonic epithelial tissues

Proximal colons from *R.gnavus*-infected and -uninfected control mice were dissected out and cut open longitudinally. After the luminal contents were washed out with ice-cold 1xPBS, the proximal colons were cut into small pieces and homogenized for total RNA isolation using TaKaRa MiniBEST Universal RNA Extraction Kit (TaKaRa, 9767). About 0.5–1 µg total RNA was reverse transcribed into first strand cDNA using PrimeScript™ 1st Strand cDNA Synthesis Kit (TaKaRa, 6110A) according to the manufacturer’s instructions. qPCR was set up using primer pairs listed in **Supplementary Table 1** and iQ™ SYBR Green Supermix (Bio-rad, 170-8884), and performed on the CFX Connect™ Real-Time System. GAPDH was used as an endogenous “housekeeping” control gene for normalization between different samples, and relative expression levels of target genes were determined using the 2^-ΔΔCt^ method (49).

### 2.7 Alnin blue and periodic acid Schiff staining

Intestinal tissue sections were processed and sectioned as described above for immunohistochemistry. An AB-PAS kit (BBI Life Science, E670107) was used to carry out alnin blue and periodic acid Schiff (PAS) staining according to the manufacturer’s instructions. Briefly, the tissue sections were washed with ultra-pure water, then stained with 1% alnin blue, followed by oxidization with the periodic acid solution and stained with Schiff reagent. Nuclei were stained with hematoxylin, followed by the Scott’s blue solution. Finally, the slides were mounted with neutral balsam mounting medium (Sangon Biotech, E675007) and imaged.

### 2.8 ELISA assays for IL-25 released from the small intestinal villi and proximal colonic epithelia

We further optimized a previously reported ELISA method to more sensitively measure stimuli-elicited IL-25 released from the intestinal samples (22). For the small intestinal samples, mouse small intestines were cut open longitudinally, washed with sterilized ice-cold 1xPBS for over 20 minutes. The intestinal villi were scraped with a glass slide, collected, and washed by centrifugation, and resuspended in 1x DPBS containing the P/S antibiotics (100 U/ml penicillin and 100 µg/ml streptomycin). The cell suspensions were washed two more times with the antibiotics-containing 1x DPBS, and then passed through a 70-µm cell strainer. The filtrates were centrifuged and the cell pellets were resuspended in Advanced DMEM/F12 supplemented with 10 mM HEPES, the antibiotics and 1 x GlutaMAX (Gibco, 35050061). The cell suspension then was aliquoted to a 24-well plate with each well containing 1x10^7^ cells, and cultured at 37°C and 5% CO_2_ for 10 h for the IL-25 ELISA assays.

The proximal colonic segments were washed with ice-cold 1xPBS, cut into 3-5 mm^2^ pieces, washed in the antibiotics-containing 1xPBS for at least 15 times and then dissociated in 1x DPBS containing 2.5 mM EDTA at 37 °C for 40 minutes, which was terminated by adding DMEM medium to the culture mixtures. The volumes of the mixtures were increased by adding additional antibiotics-containing 1x DPBS, which then were vigorously shaken a few times to release crypt cells from the epithelium before being filtered through a 70-µm cell strainer. The filtrates were centrifuged and resuspended in Advanced DMEM/F12 supplemented with 10 mM HEPES, the antibiotics and 1 x GlutaMAX as described above for the small intestinal samples. About 3 x 10^6^ cells were seeded in each well of a 24-well plate and cultured at 37°C and 5% CO_2_ for 10 h for IL-25 ELISA assays.

During the culture of the cell mixtures, stimuli such as the *R. gnavus* or *T. spiralis* lysates were added to the cells in the wells of 24-well plates. And to determine the inhibitory effect, the bitter taste receptor inhibitor allyl isothiocyanate (AITC) (Sigma-Aldrich, 57-06-7) at 3 mM, G protein subunit inhibitor Gallein (APExBIO, B7271) at 100 µM, and PLCβ2 inhibitor U73122 (Sigma-Aldrich, U6756) at 30 µM were added to the wells 1 h before the corresponding stimuli were added to the same wells. Ten hours later, cell culture in each well was spun down and the supernatant was transferred to a fresh tube, to which 1x protease inhibitor (cOmplete™, EDTA-free Protease Inhibitor Cocktail, 11873580001, Roche) was added. And the samples were used for ELISA assays or stored at -80°C until use. The amount of the released IL-25 was determined using Mouse IL-17E DuoSet ELISA kits (R&D Systems, DY1399) according to the manufacturer’s instructions. If IL-25 concentration in the sample was below this kit’s low limit of 62.5 pg/ml, another Mouse IL-17E kit with a range from 3.9 to 250 pg/ml (Thermo Fisher Scientific, BMS6046), was used.

### 2.9 Statistical analysis

The numbers of tuft cells per villus in the small intestinal segments of *R. gnavus*-infected or uninfected mice were counted while the numbers of tuft cells and goblet cells per crypt in the proximal, mid and distal colons were quantified as well following the previously described methods (26, 50, 51). The size of goblet cells and the fluorescence intensities of immunostaining images were determined using the ImageJ software. The percentages of TUNEL-positive apoptotic cells and the cleaved caspase 3, 8 or 9- positive cells in the proximal colon of *R. gnavus*-infected or uninfected mice were counted, and data from these experiments as well as from the qPCR and ELISA assays were obtained from at least three independent experiments, each experiments including at least three biological replicates. Data from at least three independent experiments were averaged and presented as means ± SD. Shapiro-Wilk normal distribution was tested. F test was performed for the homogeneity of variance before pairwise comparisons were carried out. Unpaired two-tailed Student’s t-tests were performed. For data of three or more groups, one-way ANOVA was tested using GraphPad Prism 8 software. p values <0.05 were considered statistically significant.

## 3 Results

### 3.1 *Ruminococcus gnavus* infection induces tuft cell proliferation in the proximal colon

*R. gnavus* has been reported to be particularly abundant in the gastrointestinal tract of some Covid-19 patients, and possibly associated with diarrhea, asthma, and other symptoms (5) (**Supplementary Figure 1**). To understand how *R. gnavus* interacts with host cells in the intestine, we set out to determine whether *R. gnavus* infection can regulate the number of tuft cells, a rare type of cells that have recently been discovered to detect a number of pathogens, activating innate immune responses and triggering its own hyperplasia (33).

**Figure 1:**
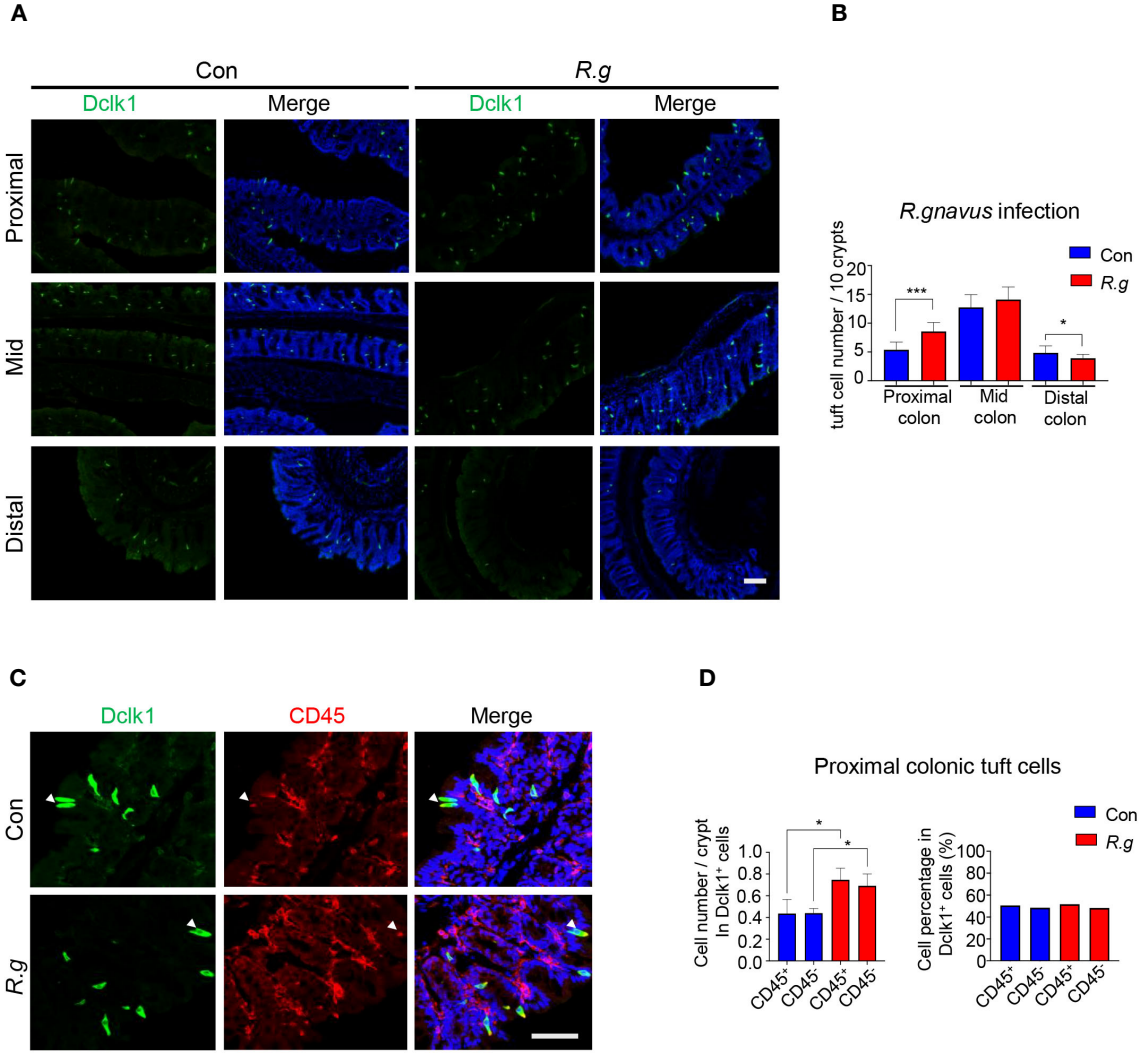
*R. gnavus* infection induces tuft cell expansion in the proximal colon. **(A)** Representative images of anti-Dclk1 antibody staining (green) on the proximal, mid and distal colon of *R. ganvus*-uninfected control (Con) and -infected (R.g) mice. Scale bar: 100 µm. **(B)** Statistical analysis of tuft cell numbers indicates that there were significantly more tuft cells in the infected proximal colon than the corresponding control, but no significant differences in the mid colon, and somewhat reduction in the distal colon. The numbers of tuft cells were counted from 100 crypts from each mouse, and three mice per group were used, and data are presented as means ± SD per 10 crypts. The data were tested for normal distribution and unpaired two-tailed Student’s t-tests were performed. *P<0.05; ***P<0.001. **(C)** Representative images of anti-Dclk1 and anti-CD45 antibody double immunostaining of the proximal colon from control (Con) and *R. ganvus* infected (R.g) mice. The tissues were stained with Dclk1 antibody (green), CD45 antibody(red) and DAPI (blue). Scale bar: 100 µm. **(D)** Statistical analysis of tuft cell numbers per crypt indicates that the numbers of both CD45^-^ subtype I and CD45^+^ subtype II tuft cells were significantly increased in the *R. gnavus*-infected proximal colon comparing with their corresponding *R. ganvus*-uninfected controls but their percentages among all proximal colonic tuft cells did not change following *R. gnavus* infection. The number of CD45^+^ and CD45^-^ tuft cells were counted from about 60 crypts of three mice. Data were tested for normal distribution and are shown as means ± SD. Unpaired two-tailed Student’s t-tests were performed. *P<0.05.

Following two doses of *R. gnavus* oral gavage on days 0 and 7, the fecal samples of both infected and uninfected control mice were collected on day 21 for DNA extraction and qPCR assays to determine the abundancy of *R. gnavus* using the PCR primers for the 16S rRNA genes, and then the mice were sacrificed, and their intestines were fixed, sectioned and immunostained with an antibody against a tuft cell marker protein Dclk1. Results showed that the abundancy of *R. gnavus* was significantly increased in the fecal samples following the oral gavage, indicating the successful inoculation of the microbe (**Supplementary Figure 2A**), and further, the number of tuft cells in the villi of the small intestine: duodenum, jejunum and ileum, did not change following *R. gnavus* inoculation in comparison with the uninfected control (**Supplementary Figures 2B, C**). In contrast, the number of tuft cells was significantly augmented in the proximal colon of *R. gnavus*-infected mice comparing with that of the uninfected wild-type control mice whereas the number was not significantly altered in the mid colon, and somewhat decreased in the distal colon (**Figures 1A, B**, **Supplementary Figure 2D**). Thereafter, this study would be mostly focused on the proximal colon although the effect of *R. gnavus* infection on the distal colonic tuft cells also warranted further studies.

Previous research has categorized tuft cells into two subtypes: I and II (28, 36). To determine which subtype undergoes expansion in response to *R. gnavus* infection, we used an antibody against CD45, a biomarker for subtype II tuft cells. Quantitative analyses showed that the numbers of both subtypes of tuft cells increased proportionally following *R. gnavus* infection, and the percentages of these two cell subtypes did not change (**Figures 1C, D**), indicating that *R. gnavus* infection can equally effectively evoke proliferation of these two tuft cell subtypes.

### 3.2 *Ruminococcus gnavus* may activate Tas2r receptors and release IL-25 from tuft cells

**Figure 2:**
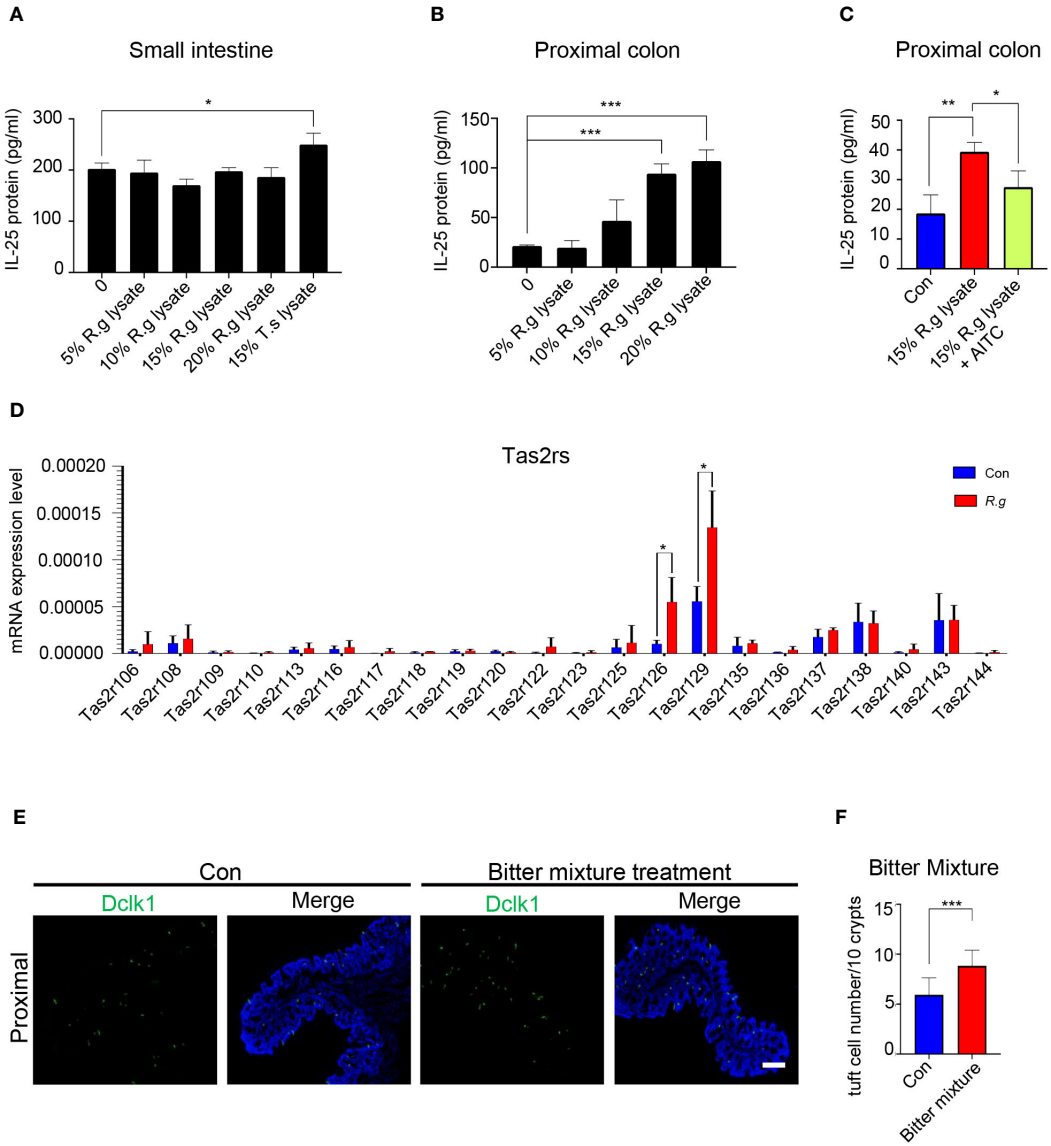
*R. gnavus* infection activates Tas2rs to release IL-25 and induce tuft cell expansion. **(A)** ELISA results indicate that a series of *R. gnavus* lysate (R.g lysate) with the highest concentration of 20% of the original solution was unable to stimulate the small intestinal epithelial cells to release significantly more IL-25 whereas the positive control, 15% *T. spiralis* lysate (T.s lysate) did. Data were obtained from three independent ELISA experiments, and each experiment included three replicates for each data point. Data (means ± SD) were tested for normal distribution and One-way ANOVA tests were performed. **(B)** *R. gnavus* lysate evoked IL-25 release from the proximal colonic epithelial cells in a dose-dependent fashion and statistically significantly more IL-25 was detected with 15% and 20% *R. gnavus* lysates. Data were obtained from three independent ELISA experiments, and each experiment included three replicates for each data point. Data (means ± SD) were tested for normal distribution and One-way ANOVA tests were performed. **(C)** IL-25 ELISA showed that the bitter receptor inhibitor AITC can significantly reduce *R.gnavus* lysate-evoked IL-25 release from the proximal colonic epithelial cells. Data were obtained from three independent ELISA experiments, and each experiment included three replicates for each data point. Data (means ± SD) were tested for normal distribution and unpaired student t tests were performed. **(D)** qRT-PCR analysis of expression of 35 mouse Tas2r genes in the *R. gnavus*-infected proximal colon (*R.g*) versus uninfected control (Con). The results showed that Tas2r126 and Tas2r129 were significantly upregulated. Data were obtained from three independent qPCR experiments, and each experiment included three replicates for each data point. Data (means ± SD) were tested for normal distribution and unpaired student t tests were performed. **(E)** Representative images of anti-Dclk1 staining (green) of control (Con) and bitter mixture-treated proximal colon. Scale bar: 100 μm. **(F)** Statistical analyses indicate that bitter mixture treatment significantly increased the number of tuft cells per 10 crypts in the proximal colon. Data were obtained from 100 crypts of three mice per group, are presented as means ± SD. Normal distributions of the data were tested and unpaired student t tests were performed. *P<0.05; **P<0.01; ***P<0.001.

Activation of tuft cells from the small intestine and other tissues often results in the release of the cytokine interleukin 25 (IL-25) (22, 25, 27). To determine whether *R. gnavus* engenders the same effect on the intestinal tuft cells, we performed ELISA assays to evaluate the release of IL-25 from the intestinal cells (**Supplementary Figure 3A**). A series of *R. gnavus* lysate concentrations were used to stimulate small intestinal tuft cells, and results showed that even the highest concentration tested, i.e., 25% of *R. gnavus* lysate, did not evoke any detectable increase in IL-25 release whereas a positive control, i.e., the lysate of the parasitic helminth *T. spiralis*, indeed induced the release of IL-25 from the small intestinal epithelial cells (**Figure 2A**). On the contrary, ELISA data showed that both 15% and 20% *R. gnavus* lysate were able to stimulate the proximal colonic epithelial cells to release significantly more IL-25 than control while 10% *R. gnavus* lysate seemed to be able to evoke a perceivable amount of IL-25 as well (**Figure 2B**). Given that microbes synthesize abundant succinic acid, which can activate small intestinal tuft cells to release IL-25 (21), we also performed ELISA assays with succinic acid acting on the proximal colonic samples. The results showed that succinic acid was unable to induce the proximal colonic cells to release IL-25 (**Supplementary Figure 3B**), implying that *R. gnavus* lysate-elicited IL-25 was not mediated by the succinate receptor (Sucnr1), which is corroborated by recent reports indicating that Sucnr1 is not expressed in the proximal colonic epithelial cells (52, 53).

To determine whether Tas2r receptors are involved in the *R. gnavus* lysate-induced IL-25 release, we applied the Tas2r inhibitor allyl isothiocyanate (AITC) to the ELISA assay. And the results showed that AITC was able to partially inhibit the IL-25 release (**Figure 2C**), suggesting that *R. gnavus* lysate may activate Tas2r receptors present on the proximal colonic tuft cells. Since AITC can also act on the Trp A1 ion channel (54), our reanalysis of available single cell RNAseq data (55) showed that Trp A1 was hardly expressed in few transit amplifying cells in the proximal colon, but not in tuft cells that producing IL-25 (**Supplementary Figure 4**), suggesting that the inhibitory effect of AITC on IL-25 release is likely through inhibiting the activity of Tas2rs expressed on tuft cells.

In an attempt to determine which Tas2rs may respond to *R. gnavus* lysate, quantitative reverse transcription-PCR was performed to identify Tas2rs of which the expression was upregulated along with tuft cell expansion following *R. gnavus* infection; and the results indicated that while the expression of a number of Tas2rs were detectable, Tas2r126 and Tas2r129 were significantly upregulated in the *R. gnavus*-infected proximal colon (**Figure 2D**), supporting the notion that Tas2r receptors may contribute to sensing *R.gnavus* infection. Since Tas2r126 can be activated by salicin while Tas2r129 is still an orphan receptor (56, 57), we prepared a bitter tastant mixture of salicin with another promiscuous bitter compound, quinine, which can stimulate multiple Tas2rs (58). Anal administration of this mixture to the mice was indeed able to increase the number of tuft cells in the proximal colon (**Figures 2E, F**), supporting Tas2rs’ possible roles in the response of the proximal colonic tuft cells to *R. gnavus* infection.

### 3.3 *Ruminococcus gnavus* activates the gustatory signaling pathway

Taste signaling proteins have been shown to play important roles in tuft cells’ detection and response to many pathogens. To determine whether these proteins also mediate colonic tuft cells’ interactions with *R. gnavus*, we set out to characterize the possible roles of three key signaling proteins: heterotrimeric G protein subunit Gγ13, phospholipase Cβ2 (PLCβ2) and transient receptor potential ion channel Trpm5.

**Figure 3:**
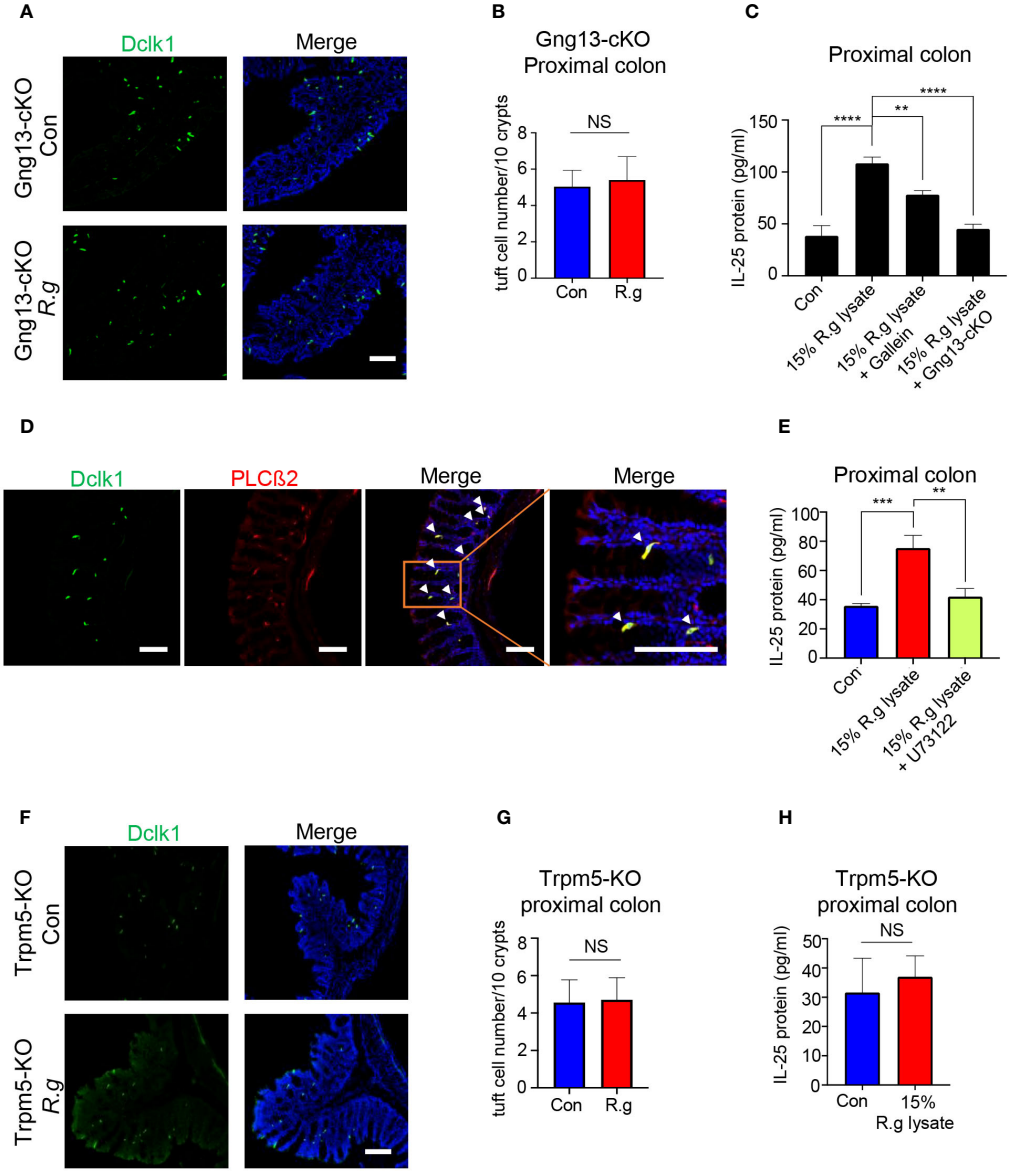
Taste signaling proteins Gγ13, PLCβ2 and Trpm5 are critical to tuft cell expansion and IL-25 release. **(A)** Representative images of anti-Dclk1 antibody staining on the proximal colon sections from *R. ganvus* -uninfected (Con) and infected (R.g) Gng13-cKO mice. The tissues were stained with Dclk1 antibody (green) and DAPI (blue). Scale bar: 100 μm. **(B)** Statistical analysis of tuft cell numbers per 10 crypts showed no significant difference in the proximal colons with or without *R. gnavus* infection. Data are shown as means ± SD obtained from 100 crypts of three mice per group. The Shapiro-Wilk test was performed to test the normal distribution of the data, and Mann-Whitney test was performed to comparatively analyze the data between the two groups. **(C)** IL-25 ELISA showed that 15% *R. gnavus* lysate (R.g lysate) could significantly elevate IL-25 release from WT proximal colonic epithelial cells, whereas the Gβγ subunit inhibitor Gallein or Gng13 conditional knockout (Gng13-cKO) significantly inhibited IL-25 release. Data were obtained from three independent ELISA experiments, each experiment including three replicates for each data point. Data (means ± SD) were tested for normal distribution and One-way ANOVA tests were performed. **(D)** Double immunostaining with anti-Dclk1and anti-PLCβ2 antibodies showed the colocalization of Dclk1 and PLCβ2 to most of the proximal colonic tuft cells. Arrowheads indicate the double-stained cells. Scale bar: 100 μm. **(E)** IL-25 ELISA results indicate the significant suppression of 15% *R. gnavus* lysate-induced IL-25 release from the proximal colonic cells by the PLCβ2 inhibitor U73122. Data were obtained from three independent ELISA experiments, each experiment including three replicates for each data point. Data (means ± SD) were tested for normal distribution and One-way ANOVA tests were performed. **(F, G)** Representative images of anti-Dclk1 antibody staining on the *R. ganvus*-uninfected (Con) and -infected (R.g) Trpm5-KO proximal colon sections. Statistical analysis showed no significant changes in the number of tuft cells per 10 crypts. Data were obtained from 100 crypts of three mice per group, are presented as means ± SD. Normal distributions of the data were tested and unpaired student t tests were performed. Scale bar: 100 μm. **(H)** IL-25 ELISA results showed that 15% *R. gnavus* lysate (R.g lysate) was unable to elicit more IL-25 release from Trpm-KO colonic epithelial cells. Data were obtained from three independent ELISA experiments, each experiment including three replicates for each data point. Data (means ± SD) were tested for normal distribution. Unpaired Student’s t-tests were performed. **P<0.01; ***P<0.001, ****P<0.0001. ns, not significant.

Bioinformatical reanalysis of the proximal colonic single-cell RNAseq data (52) indicated that both choline acetyltransferase (ChAT) and Gng13 were co-expressed in the proximal colonic tuft cells (**Supplementary Figures 5A, B**). Immunostaining with the Dclk1 antibody on the ChAT-Ai9 proximal colonic tissue sections indicated that about 83.6% of Dclk1-positive tuft cells were also tdTomato-positive (**Supplementary Figures 5C, D**) while immunostaining with the CD45 antibody showed that about 58.2% and 41.8% of ChAT-Ai9+ cells were CD45- and CD45+ subtypes I and II tuft cells, respectively (**Supplementary Figures 5E, F**), suggesting that ChAT is expressed in both subtypes of tuft cells. The Gng13 conditional knockout (Gng13-cKO) mice in the proximal colonic tuft cells were generated by crossing ChAT-Cre mice with Gng13^Flox/Flox^ mice and quantitative RT-PCR data showed a significantly reduced expression of Gng13 transcripts in the Gng13-cKO proximal colon (**Supplementary Figure 5G**). To test whether the Gng13-encoded Gγ13 subunit contributes to the *R. gnavus*-induced tuft cell expansion, qRT-PCR and immunostaining were performed on the Gng13-cKO mice following the *R. gnavus* inoculation. Results showed that Gng13-cKO abolished the *R. gnavus*-induced increase in Dclk1 expression (**Supplementary Figure 5H**) while the increase in the number of tuft cells in the *R. gnavus*-infected proximal colon was insignificant comparing with the uninfected control (**Figures 3A, B**, **Supplementary Figure 6A**). To assess the G protein βγ subunits’ role in IL-25 release from tuft cells, we carried out IL-25 ELISA assay on WT colonic cells in the presence of G protein βγ subunits’ inhibitor Gallein, or on Gng13-cKO colonic cells. The results indicated that Gallein was able to partially inhibit IL-25 release from WT colonic cells whereas Gng13 conditional knockout also resulted in a significant reduction in IL-25 release (**Figure 3C**).

PLCβ2 is known to be an effector enzyme that generates the second messengers upon activation by the Gβγ moiety in taste receptor cells as well as tuft cells in some tissues (22, 59). Reanalysis of the single proximal colonic cell RNAseq data indicated the expression of PLCβ2 in tuft cells (**Supplementary Figure 6B**) while double immunostaining showed that PLCβ2 is indeed colocalized with Dclk1 (**Figure 3D**). And IL-25 ELISA assays indicated that the PLCβ2 inhibitor U73122 significantly suppressed the IL-25 release from the proximal colonic tuft cells (**Figure 3E**).

The transient receptor potential ion channel Trpm5 is a key signaling protein in bitter, sweet and umami taste transduction in taste buds, and also critical to the detection and response of small intestinal tuft cells to pathogens (22, 25). Analyses of single colonic cell RNAseq data from the showed the expression of Trpm5 in tuft cells, and immunostaining with Dclk1 antibody on Trpm5^+/-^ heterozygous tissue sections also showed the colocalization of Dclk1 with lacZ staining to tuft cells (**Supplementary Figures 6C, D**). Further analyses indicated that *R. gnavus* infection did not increase Dclk1 transcripts or the number of Trpm5-expressing tuft cells in the proximal colon (**Figures 3F, G**, **Supplementary Figure 5H**), and that *R. gnavus* lysate was unable to induce significantly more IL-25 release from the Trpm5-KO proximal colonic cells comparing with the negative control sample (**Figure 3H**).

### 3.4 *Ruminococcus gnavus* infection does not increase the number or size of goblet cells

**Figure 4:**
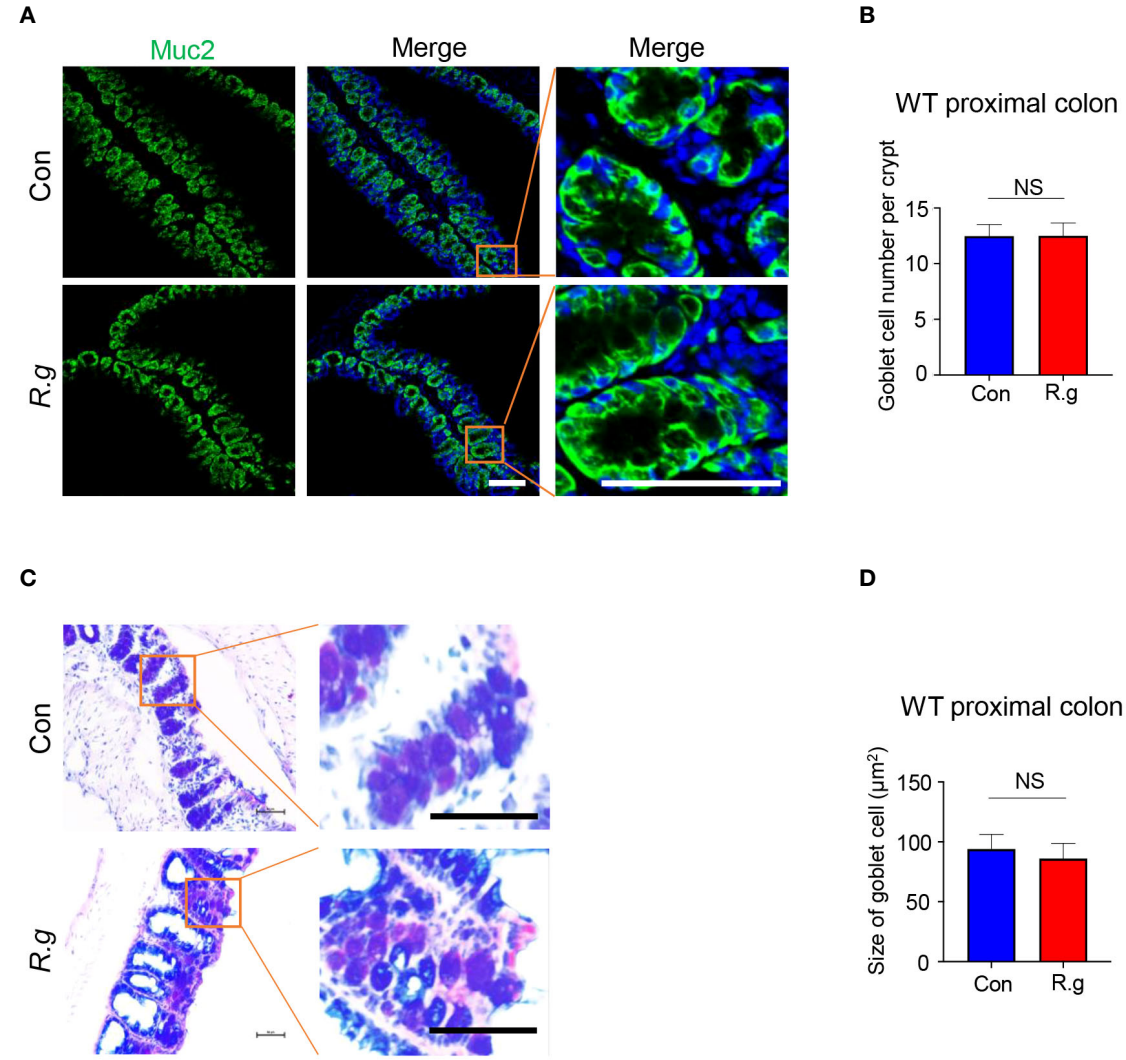
*R. gnavus* infection does not elicit goblet cell hyperplasia or an increase in its size. **(A, B)** Immunostaining of *R. gnavus*-uninfected (Con) and -infected (*R.g*) proximal colonic sections with an antibody to the goblet cell marker mucin 2 (Muc2). No significant change was found in the number of goblet cells between control and *R. gnavus*-infected proximal colons. Data were obtained from 100 crypts of three mice per group, and presented as means ± SD. Normal distributions of the data were tested and unpaired student t tests were performed. Scale bar: 100 μm. **(C, D)** AB-PAS staining of *R. gnavus*-uninfected (Con) and -infected (R.g) proximal colonic sections. No significant change was found in the size of goblet cells between control and *R. gnavus-*infected proximal colons. Data were obtained from 200 crypts of three mice per group, are presented as means ± SD. Normal distributions of the data were tested and unpaired student t tests were performed. Scale bar: 50 μm. ns, not significant.

To determine whether *R. gnavus* infection also affects goblet cells’ gene expression, cell turnover or physiological activity, we carried out quantitative RT-PCR, immunohistochemistry and alnin blue and periodic acid Schiff (AB-PAS) staining (**Figure 4**, **Supplementary Figure 7**). qRT-PCR results indicated that *R. gnavus* infection did not significantly increase the expression of some goblet cells’ marker genes: *Spink4*, *Muc2*, *Txndc5*, *Fcgbp* and *Spdef* (**Supplementary Figure 7A**) whereas immunostaining with an anti-Muc2 antibody showed no significant increase in the number of goblet cells either following *R.gnavus* infection (**Figures 4A, B**, **Supplementary Figure 7B**). Neither did AB-PAS staining show any changes in goblet cell size (**Figures 4C, D**).

### 3.5 *Ruminococcus gnavus* infection and taste signaling proteins regulate apoptotic gene expression and cell death

**Figure 5:**
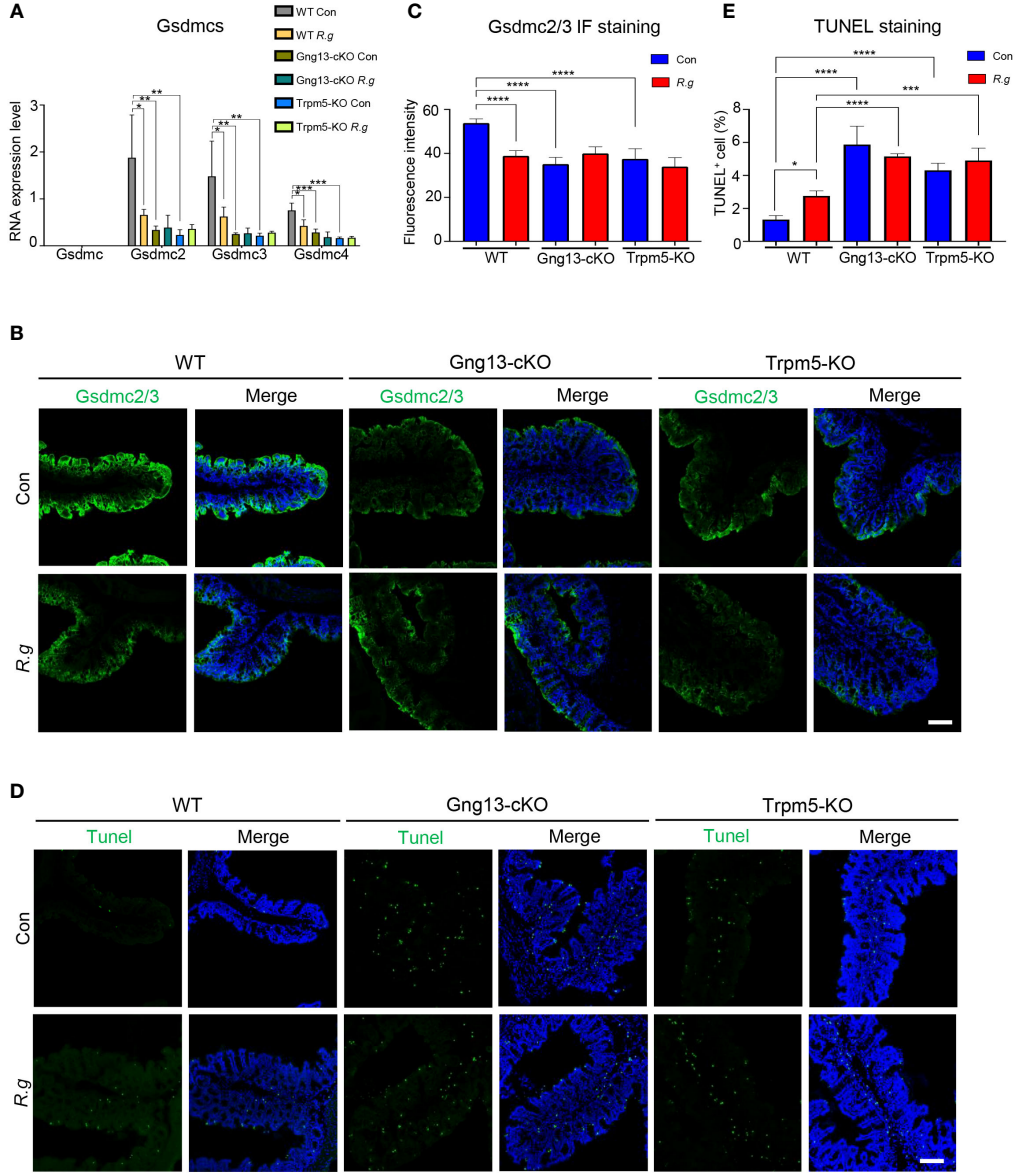
*R. gnavus* and taste signaling proteins regulate gasdermin expression. **(A)** qRT-PCR analysis indicates that while Gsdmc expression was not detectable in the proximal colon, Gsdmc2, Gsdmc3 and Gsdmc4 were abundantly expressed (WT Con). *R. gnavus* infection (WT R.g) significantly reduced Gsdmc2, Gsdmc3 and Gsdmc4 expression comparing with corresponding WT control (WT Con). Gng13-KO (Gng13-cKO Con) and Trpm5-KO (Trpm5-KO Con) significantly decreased all these gasdermins’ expression comparing with their corresponding WT control (WT Con). *R. gnavus* infection did not further reduce these gasdermins’ expression in Gng13-cKO or Trpm5-KO mice. qPCR data were obtained from three replicates and presented as means ± SD, and tested for normal distribution. One-way ANOVA and Mann Whitney tests were performed. **(B, C)** Immunostaining with an antibody to both Gsdmc2 and Gsdmc3 (Gsdmc2/3) showed that Gsdmc2/3 immunosignal was significantly reduced in *R. gnavus*-infected WT proximal colon (WT R.g) comparing with uninfected WT control (WT Con). Gng13-cKO and Trpm5-KO also showed a decrease in Gsdmc2/3 immunosignal intensity (Gng13-cKO Con, Trpm5-KO Con) comparing with that of WT control (WT Con). *R. gnavus* infection did not further reduce Gsdmc2/3 immunosignal in Gng13-cKO or Trpm5-KO proximal colons (Gng13-cKO R.g vs. Gng13-cKO Con; Trpm5-KO R.g vs. Trpm5-KO Con). Data were obtained from 15 tissue sections of three mice per group, and presented as means ± SD. The normal distributions of the data were tested and One-way ANOVA tests were performed. Scale bar: 100 μm. **(D, E)** TUNEL assays on the proximal colon sections show that TUNEL-positive cells were increased in *R. gnavus*-infected sections (WT R.g) than in the uninfected control (WT Con). Bothe uninfected Gng13-KO and Trpm5-KO (Gng13-cKO Con, Trpm5-KO Con) had significantly more TUNEL-positive cells than WT control (WT Con). *R. gnavus* infection did not further increase TUNEL-positive cells in the two knockout colons (Gng13-cKO R.g vs. Gng13-cKO Con, Trpm5-KO R.g vs. Trpm5-KO Con). Data were obtained from 15 tissue sections of three mice per group, and presented as means ± SD. The normal distributions of the data were tested and One-way ANOVA tests were performed. Scale bar: 100 μm. *P<0.05; **P<0.01; ***P<0.001; ****P<0.0001.

Infection of pathogens can lead to intestinal cell death (42). Analysis of the single-cell RNAseq data showed abundant expression of pyroptotic genes, including Gasdermin C2, C3, and C4 (*Gsdmc2*, *Gsdmc3*, *Gsdmc4*) in a number of colonic cell types (**Supplementary Figure 8**) (52). To determine whether *R. gnavus* infection, Gγ13 or Trpm5 contributes to pyroptotic gene expression regulation, we first performed qRT-PCR and found that *Gsdmc* was indeed not expressed in the proximal colon (**Figure 5A**), which is consistent with the scRNAseq data (**Supplementary Figure 8A**), while the expression of *Gsdmc2*, *Gsdmc3* and *Gsdmc4* was strong in WT proximal colons (**Figure 5A**). *R. gnavus* infection, however, significantly reduced *Gsdmc2*, *Gsdmc3* and *Gsdmc4* expression (**Figure 5A**). Conditional knockout of Gng13 or nullification of Trpm5 greatly reduced the expression of all *Gsdmc2*, *Gsdmc3* and *Gsdmc4* genes comparing with WT control whereas *R. gnavus* infection did not further reduce these genes’ expression comparing with their corresponding uninfected controls.

To assess the protein expression levels of these gasdermins in the proximal colon, we performed immunohistochemistry with an antibody to both Gsdmc2 and Gsdmc3. And the results showed that fluorescent signal was strongest on the proximal colonic epithelium from the uninfected WT control mice while *R. gnavus* infection significantly decreased Gsdmc2/3 expression (**Figures 5B, C**, **Supplementary Figure 9A**). Both Gng13-cKO and Trpm5-KO uninfected colonic epithelia had much reduced Gsdmc2/3 signal intensity comparing with that of the uninfected WT, and *R. gnavus* infection did not further reduce the signal intensity in these mutant mice comparing with their respective uninfected knockout controls.

Given that *R. gnavus* infection and taste signaling proteins altered gasdermin expression, we then set out to determine how the infection affects cell death in the proximal colon. TUNEL assays were performed, which revealed that *R. gnavus* infection significantly increased the number of TUNEL-positive cells in WT proximal colon, whereas the *R. gnavus*-uninfected Gng13-cKO and Trpm5-KO mice showed significantly more TUNEL-positive cells than *R. gnavus*-uninfected WT (**Figures 5D, E**, **Supplementary Figure 9B**), and these TUNEL-positive cells were located at both the top and bottom of the crypts, suggesting that they include both mature colonocytes and stem cells, which is consistent with the spatial transcriptome analysis of the single-cell RNAseq data (**Supplementary Figure 9C**) (52, 60, 61). Comparing with *R. gnavus*-infected WT mice, *R. gnavus*-infected Gng13-cKO and Trpm5-KO mice displayed significantly more TUNEL-positive cells (**Figures 5D, E**).

### 3.6 *Ruminococcus gnavus* infection and taste signaling proteins regulate caspase activation

**Figure 6:**
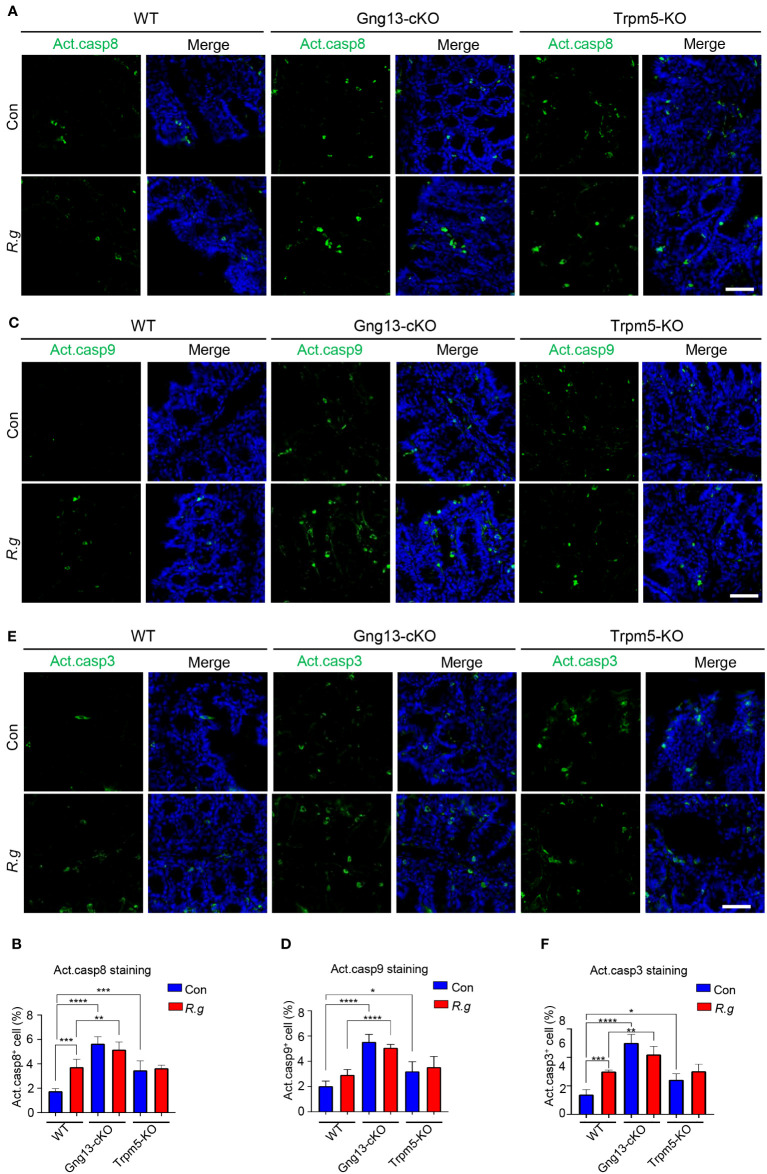
*R. gnavus* infection and taste signaling proteins regulate cleavage of caspases 8, 9 and 3. **(A, B)** Immunostaining of the proximal colons with an antibody to cleaved active caspase 8 (Act. casp8) showed that *R. gnavus* infection increased the number active caspase 8-positive cells in WT mice (WT R.g vs. WT Con), but not in the Gng13-cKO or Trpm5-KO mice (Gng13-cKO R.g vs. Gng13-cKO Con, Trpm5-KO R.g vs. Trpm5-KO Con). Uninfected Gng13-cKO and Trpm5-KO mice had significantly more active caspase 8-positive cells than WT control (Gng13-cKO Con vs. WT Con, Trpm5-KO Con vs. WT con). However, only *R. gnavus*-infected Gng13-cKO but not infected Trpm5-KO mice had more active caspase 8-positive cells than infected WT control (Gng13-cKO R.g vs. WT R.g, Trpm5-KO R.g vs. WT R.g). **(C–F)** Similar patterns to those of the above active caspase 8 were found for active caspases 9 and 3 except that *R. gnavus* infection did not significantly increase the number of active caspase 9-positive cells in WT mice (WT R.g vs. WT Con). Data were obtained from 15 tissue sections of three mice per group, and presented as means ± SD. The normal distributions of the data were tested and One-way ANOVA tests were performed. Scale bar: 100 μm. *P<0.05; **P<0.01; ***P<0.001; ****P<0.0001.

Apoptosis is known to be activated via two signaling pathways: extrinsic and intrinsic (62). The former is triggered by extracellular death factors, leading to the cleavage of procaspase 8 into active caspase 8 while the latter is by intracellular factors such as genotoxic agents, leading to the modification of procaspase 9 into active caspase 9. Both active caspases 8 and 9 converge on cleaving procaspase 3 into active caspase 3, which degrade cellular proteins, resulting in eventual apoptosis (62). To determine whether both extrinsic and intrinsic signaling pathways contribute to *R. gnavus*-induced apoptosis, we performed immunohistochemistry with antibodies against each of these three activated caspases: 3, 8 and 9. Results showed that *R. gnavus* infection indeed activated significantly more caspases 3 and 8 but not 9 in the proximal colon (**Figure 6**, **Supplementary Figure 10**). The uninfected Gng13-cKO and Trpm5-KO mice displayed significantly more activated caspase 3, 8 or 9-positive cells than uninfected WT mice whereas the infection did not significantly alter the number of activated caspases 3, 8 or 9-positive cells in either the Gng13-cKO or Trpm5-KO proximal colon. However, only in the infected Gng13-cKO, but not in the infected Trpm5-KO, more proximal colonic cells with activated caspases 3, 8 and 9 were found than in the infected WT control mice (**Figure 6**).

## 4 Discussion

*R. gnavus* is a type of pathogens that have been found in many patients of different diseases, from chronic bowel disorders to Covid-19 infection (3–6, 9, 11). The molecular and cellular mechanisms underlying its pathogenesis are yet to be fully understood.

*R. gnavus* normally colonizes in the large intestine. We found that oral administration of *R. gnavus* induced the expansion of tuft cells in the proximal colon, but did not affect tuft cell generation in the mid colon, and somewhat suppressed tuft cell generation in the distal colon (**Figure 1**). These results indicate that the proximal and distal colonic cells can detect and respond to *R. gnavus*, leading to different outcomes, while the mid colon is unable to or its response does not involve tuft cells. It is interesting to understand why *R. gnavus* infection engenders opposite changes in tuft cell numbers in the proximal versus distal colons. One possible reason is that *R. gnavus* mostly colonizes in the proximal colon (63), and another is that tuft cells in the proximal and distal colons are very different in terms of both gene expression profiles and perhaps physiological functions (**Supplementary Figure 11**), sensing and responding differently. However, further studies are needed to definitively address this differential effect.

Tuft cells have been classified into at least two subtypes based on their gene expression profiles, receptor expression patterns and signal transduction pathways (28, 36). How the turnover of each subtype is regulated is not known yet although the transcription factor Atoh1 has been shown to promote the progenitor cells to proliferate and differentiate into tuft cells (64). *R. gnavus* infection increased the cell numbers of both subtypes in an equal proportion, suggesting that active components from *R. gnavus* can stimulate both subtypes to proliferate at a similar rate. Further investigation is warranted to identify the active molecules from the microbe and their corresponding receptors on tuft cells.

IL-25 is a critical cytokine to small intestinal tuft cells’ response to invading pathogens (33). We found that *R. gnavus* lysate can also stimulate proximal colonic tuft cells to release IL-25 in a concentration-dependent manner (**Figure 2**). However, *R. gnavus* lysate could not evoke IL-25 release from the small intestinal villi whereas *T. spiralis* lysate could (**Figure 2**) (22). These results indicate that the small and large intestinal tuft cells express different sets of receptors that can sense different active components from *T. spiralis* and *R. gnavus*, respectively. Furthermore, the small intestinal tuft cells express succinate receptors and can be stimulated by succinate acid to release in IL-25 while the proximal colonic tuft cells do neither express the receptor, nor respond to succinate acid. Instead, the responses of the proximal colonic tuft cells to *R. gnavus* lysate can be inhibited by the bitter taste receptor inhibitor AITC whereas expression of Tas2r126 and Tas2r129 was upregulated along with *R. gnavus*-induced tuft cell expansion in the proximal colon. Besides, anal administration of a mixture of bitter tasting compounds of salicin and quinine could effectively elicit tuft cell expansion in the proximal colon. These lines of evidence suggest that activation of these Tas2r receptors on the proximal colonic tuft cells by bioactive molecules from *R. gnavus* results in the release of IL-25, and subsequently tuft cell expansion. However, the proximal colonic tuft cells could also utilize other G protein coupled receptors such as free fatty acid receptor, vomeronasal receptor or other yet-to-be-identified receptors, although the succinate receptor appears to be absent in these tuft cells (28, 32). Further studies are need to firmly establish the role of Tas2rs or any other receptors in the detection of *R. gnavus* infection.

As with tuft cells in many other tissues, taste signaling proteins Gγ13, PLCβ2 and Trpm5 are also important to proximal colonic tuft cells’ detection and response to *R. gnavus*. The inhibitors Gallein and U73122 to G protein βγ subunits and PLCβ2, respectively, can significantly reduce *R. gnavus* lysate-induced IL-25 release from the proximal colonic tuft cells while nullification of *Gng13* and *Trpm5* cannot only decrease IL-25 release but also abolish tuft cell expansion (**Figure 3**), suggesting that the taste signal transduction again plays a key role in the proximal colonic tuft cells’ detection and response to *R. gnavus*, leading to IL-25 release and subsequent tuft cell proliferation. Unlike in the small intestine, however, no concomitant changes in the number or size of the proximal colonic goblet cells were found following *R. gnavus* infection (**Figure 4**), indicating that the regulatory mechanisms underlying goblet cells’ proliferation and function are different in the small versus large intestines.

Gasdermins have been known to form pores on the cell membrane, leading to pyroptosis (65). A recent study, however, showed a novel role of gasderrmin Cs, that is, they can protect colonic stem cells from cell death (66). Our data indicate that *R. gnavus* infection reduced *Gsdmc2, 3* and *4* expression, and increased the number of TUNEL-positive cells and active caspases 8 and 3-positive cells but not active caspase 9 compared with those of *R. gnavus*-infected WT mice (**Figures 5**, **6**). Apoptosis can be triggered by both extrinsic and intrinsic signaling pathways, which lead to the cleavage of pro-caspases 8 and 9 into active caspases 8 and 9, respectively. The two pathways then converge on cleaving pro-caspase 3 into active caspase 3. Our results suggest that *R. gnavus* infection per se activates mostly the extrinsic pathway since only the levels of active caspases 8 and 3 but not 9 were increased (**Figure 6**). One possible extrinsic pathway can be triggered by the microbe’s metabolite glucorhamnan, which stimulates dendritic cells to secrete TNFα and act on the colonic cells (14).

In the Gng13-cKO and Trpm5-KO mice, even without *R. gnavus* infection, the expression of these three gasdermins, *Gsdmc2*, *Gsdmc3* and *Gsdmc4*, was lower than in WT control while the numbers of active caspases 3, 8 and 9 positive cells as well as TUNEL-positive cells were more than WT control, which is in agreement with the notion that gasdermins play a protective role against cell death (66). These data also indicate that the two taste signaling proteins Gγ13 and Trpm5 are critical to maintaining the colonic homeostasis by upkeeping the expression level of these gasdermins in the colonocytes and limiting both extrinsic and intrinsic pathway-mediated caspase activation as well as apoptosis to the base lines (62). Since these gene knockout animals were kept in a specific pathogen-free environment instead of a germ-free facility, some other gut microbes in these mice may still incur cell death in the proximal colon even with no oral gavage of *R. gnavus*. Interestingly, oral gavage of *R. gnavus* to Gng13-cKO or Trpm5-KO mice did not further reduce the expression of these gasdermins, nor further increase active forms of caspases 3, 8 and 9 or TUNEL-positive cells, indicating that the Gsdmc expression reached the low limits or the active forms of caspases 3, 8 and 9 or the number of TUNEL+ cells extended to the upper limits.

Comparing with the *R. gnavus*-infected WT, the infected Gng13-cKO displayed significantly more active caspases 3, 8 and 9-positive cells whereas the infected Trpm5-KO mice did not show any significant changes in the number of active caspases-positive cells (**Figure 6**), indicating that the Gng13-encoded Gγ13 may play a more important role than Trpm5 in regulating cell caspase activation in the proximal colon. This can be explained that Gγ13 is a signaling protein upstream of Trpm5 in taste signal transduction pathway. Given that tuft cells may employ multiple types of receptors, transduce signals via several transduction pathways, and release a number of output signals such as acetylcholine, prostaglandins, cysteinyl leukotrienes, and IL-25, acting on adjacent epithelial cells, immune cells or nerve endings, it is possible that Gγ13 regulates more signaling pathways and output signals than Trpm5, consequently rendering differential outcomes. Further investigations are needed to elucidate the exact molecular circuits controlled by Gγ13 and Trpm5, respectively, in regulating caspase activation in the proximal colon.

The taste signaling proteins Gγ13 and Trpm5 appear to block apoptotic cell death in the proximal colon since nullification of these two genes led to increased activation of caspases and the number of TUNEL-positive cells. This result is largely in line with our previous findings, showing that knockout of the gene for the gustatory G protein α subunit Gα-gustducin aggravated gut inflammation in an animal model for inflammatory bowel disease (67). It is possible that as in taste bud cells and small intestinal tuft cells, Gα-gustducin and Gγ13 may interact with each other in some subsets of proximal colonic tuft cells in transducing receptor-mediated signals (22, 59). In other tuft cells, however, they may play different roles. Comprehensive studies are needed to address these issues.

*R. gnavus* may play a multitude of roles in the gut. For example, it can produce such metabolites as phenethylamine and tryptamine, which can activate trace amine-associated receptor 1 (TAAR1) on the enterochromaffin cells, stimulating the biosynthesis of serotonin, causing diarrhea (4). Our studies show that *R. gnavus* infection reduces gasdermin C2/3/4 expression and increases caspase activation and apoptosis. Widespread cell death in the colon may also contribute to diarrhea, inflammation, and other symptoms, which may be alleviated by activation of the Gγ13-Trpm5 signaling pathways possibly with bitter tastants or other compounds.

## 5 Conclusion

Results from this study indicate that molecules from *R. gnavus* can stimulate the Tas2rs or other receptors-Gγ13-PLCβ2-Trpm5 molecular circuit in the proximal colonic tuft cells, resulting in the release of IL-25 and other bioactive molecules, and leading to proximal colonic tuft cell expansion (**Supplementary Figure 12**). IL-25 triggers type II innate immune responses, which help maintain gasdermin expression levels and protect the proximal colon from *R. gnavus* infection-incurred cell death. It is also possible that some bioactive molecules released from tuft cells directly act on colonic stem cells and colonocytes, protecting them from undergoing apoptosis. Novel insights gained from this study can help devise new ways to treat such disorders as allergy, asthma, diarrhea and inflammatory bowel disease that are associated with *R. gnavus* and possibly other commensal pathobionts as well.

## Data availability statement

The original contributions presented in the study are included in the article/**Supplementary Material**. Further inquiries can be directed to the corresponding authors.

## Ethics statement

The animal study was approved by the Animal Care and Use Committee of Zhejiang University. The study was conducted in accordance with the local legislation and institutional requirements.

## Author contributions

HL: Conceptualization, Formal Analysis, Methodology, Visualization, Writing – original draft. DY: Formal Analysis, Methodology, Writing – original draft, Data curation, Investigation. Y-BX: Data curation, Formal Analysis, Investigation, Methodology, Writing – original draft. Y-HL: Data curation, Formal Analysis, Investigation, Methodology, Writing – original draft. S-MG: Data curation, Formal Analysis, Investigation, Methodology, Writing – original draft. Y-YP: Data curation, Formal Analysis, Investigation, Methodology, Writing – original draft. K-FL: Investigation, Methodology, Writing – original draft. DB: Methodology, Writing – review & editing. YY: Data curation, Formal Analysis, Investigation, Methodology, Writing – original draft. S-SZ: Data curation, Formal Analysis, Investigation, Methodology, Writing – original draft. MW: Methodology, Project administration, Supervision, Writing – review & editing. RZ: Conceptualization, Formal Analysis, Funding acquisition, Supervision, Writing – review & editing. LH: Conceptualization, Data curation, Formal Analysis, Funding acquisition, Investigation, Methodology, Project administration, Resources, Supervision, Validation, Visualization, Writing – original draft, Writing – review & editing.
