# Supplementary material for: Tuft cells utilize taste signaling molecules to respond to the pathobiont microbe Ruminococcus gnavus in the proximal colon
Source: Front Immunol. 2023 Oct 25;14:1259521. doi: 10.3389/fimmu.2023.1259521 (PMC10634341; doi:10.3389/fimmu.2023.1259521)
Supplement: Supplementary file 1 [file DataSheet_1.pdf]

Supplementary Material

1 Supplementary methods

1. Fecal DNA isolation and qPCR to confirm *R. gnavus* colonization in mice

Fecal DNA was extracted from fecal samples using the Solarbio D2700 kit for Fecal Genomic DNA Extraction (Solarbio life sciences, China). Briefly, about 0.1-0.5 g fecal sample was transferred into a centrifuge tube, to which 450 µl of PBS was added. The tube was shaken vigorously for 1-2 min to dissolve the sample. About 50 µl DNA extract solution (100 mM Tris-HCl, 100 mM Na_2EDTA, 100 mM Na_3PO_4, 1.5 M NaCl, 1% CTAB) was added to the tube, which was inverted a few times to mix, and then incubated at 65 °C for 6 minutes. About 100 µl phenol /chloroform solution was added, and the tube was inverted and mixed well, then centrifuged at 12,000 rpm for 10 minutes. The supernatant was transferred to a new tube and centrifuged again. The supernatant was transferred to an adsorption column, which contained 200 µl of isopropyl alcohol. The content was mixed by pipetting several times, and centrifuged at 12,000 rpm for 1 min. The filtrate was added back to the absorption tube, and centrifuge again to remove liquid. The tube was washed twice with 500 µl rinse solution, then eluted with 50-100 µl elution buffer. The concentration of the eluted microbial DNA was determined using Nanodrop (ThermoFisher Scientific, UK). About 10 ng/µl DNA was used as a template for each qPCR reaction with iQTM SYBR Green Supermix (Bio-rad, 170-8884) on a CFX ConnectTM Real-Time System (Bio-rad). Primers of the 16S gene of *R. gnavus* strains were described previously (1).

2. Bioinformatic reanalysis of published fecal microbiome and scRNAseq datasets

Fecal microbiome data PRJNA650244 collected from 196 COVID-19 patients and 78 non-COVID-19 control adults were reanalyzed to determine microbe distribution among these subjects (Yeoh et al., 2021), GMrepo database was used to carried out LDA score analysis and *R. gnavus* flora abundance analysis (2).

Two mouse proximal colon single cell RNA sequencing (scRNAseq) datasets GSE169183 and GSE168448 and one distal colon scRNAseq dataset GSE168448 were reanalyzed to determine cell types and gene expression profiles (3, 4).'Seurat' package was used for clustering and identifying signature genes of each cluster (5). Highly variable genes were identified using the 'Find Variable Features' function. Dimensionality reduction was performed by principal component analysis with

the ‘RunPCA’ function, and top 1-20 principal components (PCs) were chosen for further analysis based on the ‘JackStrawPlot’ and ‘ElbowPlot’ functions for the proximal colon scRNAseq data. Cells were clustered using the ‘FindNeighbors’ function with a k. param of 30 and the ‘FindClusters’ function with a resolution of 0.5. Cells were then plotted using the U-MAP method. Differentially expressed genes were calculated using the ‘FindMarkers’ function with parameters min.pct = 0.1 and logfc. threshold = 0.25. R version was 4.1.3.

For the combined analyses, the mouse proximal colon spatial transcriptome dataset and single cell RNAseq dataset were extracted from GSE169749 and GSE169183, respectively, each of which was processed using the ‘Squidpy’ and ‘Scanpy’ packages, respectively (3, 6-8). The spatial localization of single cell data was achieved using the Tangram method (9). R version is 4.1.3. Python version is 3.9.

2 Supplementary Figures and Table

2.1 Supplementary Figures

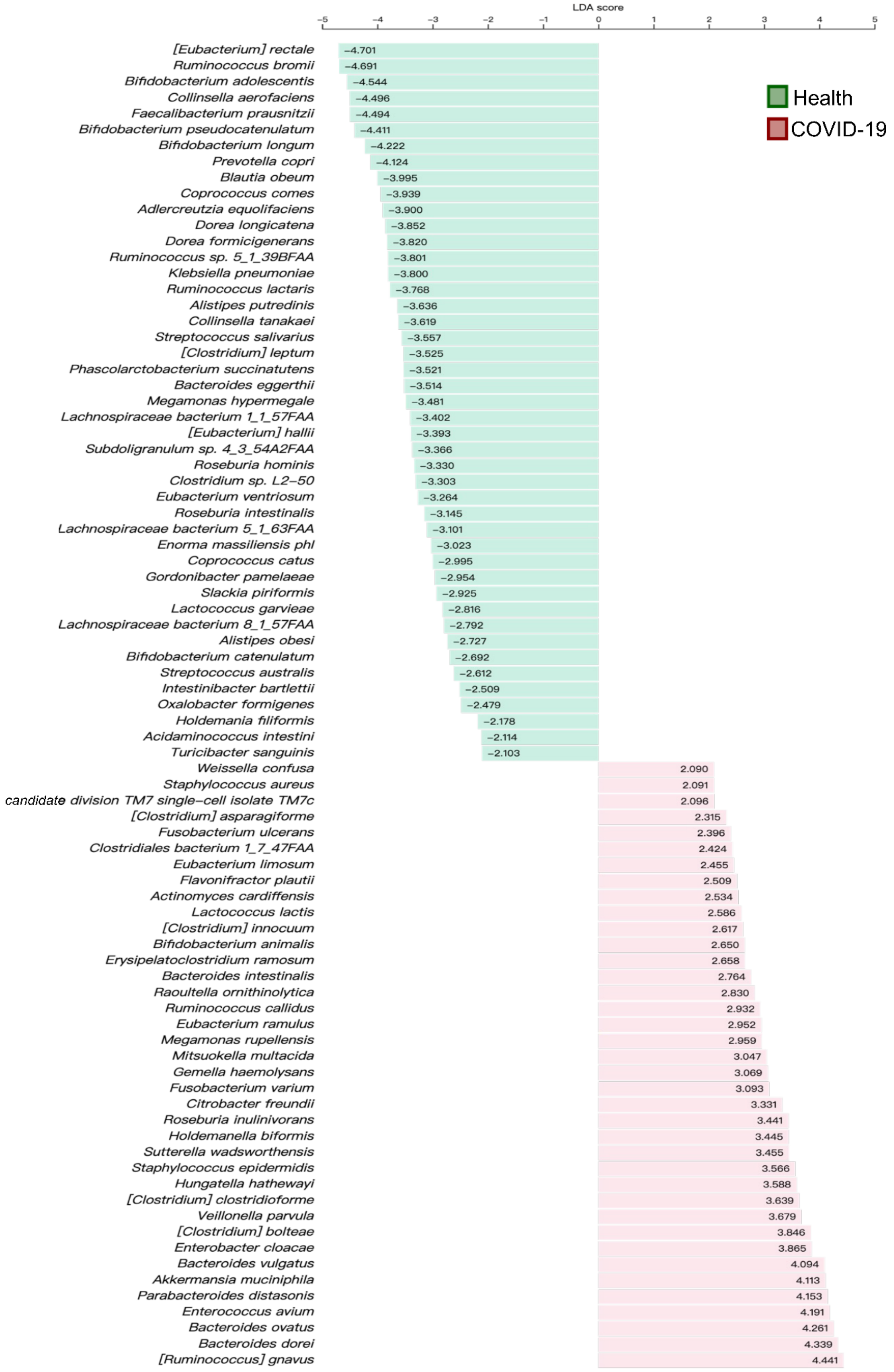
LDA score
−5
−4
−3
−2
−1
0
1
2
3
4
5
Health
COVID-19
[Eubacterium] rectale −4.701
Ruminococcus bromii −4.691
Bifidobacterium adolescentis −4.544
Collinsella aerofaciens −4.496
Faecalibacterium prausnitzii −4.494
Bifidobacterium pseudocatenulatum −4.411
Bifidobacterium longum −4.222
Prevotella copri −4.124
Blautia obeum −3.995
Coprococcus comes −3.939
Adlercreutzia equolifaciens −3.900
Dorea longicatena −3.852
Dorea formicigenerans −3.820
Ruminococcus sp. 5_1_39BFAA −3.801
Klebsiella pneumoniae −3.800
Ruminococcus lactaris −3.768
Alistipes putredinis −3.636
Collinsella tanakaei −3.619
Streptococcus salivarius −3.557
[Clostridium] leptum −3.525
Phascolarctobacterium succinatutens −3.521
Bacteroides eggerthii −3.514
Megamonas hypermegale −3.481
Lachnospiraceae bacterium 1_1_57FAA −3.402
[Eubacterium] hallii −3.393
Subdoligranulum sp. 4_3_54A2FAA −3.366
Roseburia hominis −3.330
Clostridium sp. L2−50 −3.303
Eubacterium ventriosum −3.264
Roseburia intestinalis −3.145
Lachnospiraceae bacterium 5_1_63FAA −3.101
Enorma massiliensis phl −3.023
Coprococcus catus −2.995
Gordonibacter pamelaeae −2.954
Slackia piriformis −2.925
Lactococcus garvieae −2.816
Lachnospiraceae bacterium 8_1_57FAA −2.792
Alistipes obesi −2.727
Bifidobacterium catenulatum −2.692
Streptococcus australis −2.612
Intestinibacter bartlettii −2.509
Oxalobacter formigenes −2.479
Holdemania filiformis −2.178
Acidaminococcus intestini −2.114
Turicibacter sanguinis −2.103
Weissella confusa 2.090
Staphylococcus aureus 2.091
candidate division TM7 single−cell isolate TM7c 2.096
[Clostridium] asparagiforme 2.315
Fusobacterium ulcerans 2.396
Clostridiales bacterium 1_7_47FAA 2.424
Eubacterium limosum 2.455
Flavonifractor plautii 2.509
Actinomyces cardiffensis 2.534
Lactococcus lactis 2.586
[Clostridium] innocuum 2.617
Bifidobacterium animalis 2.650
Erysipelatoclostridium ramosum 2.658
Bacteroides intestinalis 2.764
Raoultella ornithinolytica 2.830
Ruminococcus callidus 2.932
Eubacterium ramulus 2.952
Megamonas rupellensis 2.959
Mitsuokella multacida 3.047
Gemella haemolysans 3.069
Fusobacterium varium 3.093
Citrobacter freundii 3.331
Roseburia inulinivorans 3.441
Holdemanella biformis 3.445
Sutterella wadsworthensis 3.455
Staphylococcus epidermidis 3.566
Hungatella hathewayi 3.588
[Clostridium] clostridioforme 3.639
Veillonella parvula 3.679
[Clostridium] bolteae 3.846
Enterobacter cloacae 3.865
Bacteroides vulgatus 4.094
Akkermansia muciniphila 4.113
Parabacteroides distasonis 4.153
Enterococcus avium 4.191
Bacteroides ovatus 4.261
Bacteroides dorei 4.339
[Ruminococcus] gnavus 4.441

Supplementary Figure 1

Reanalysis of the published gut microbe data PRJNA650244 (10). Linear discriminant analysis (LDA) scores of fecal microbiomes indicate the relative abundance of gut microbes among the health subjects and COVID-19 patients. *R. gnavus* is the most abundant microbe in the patients (listed at the very bottom).

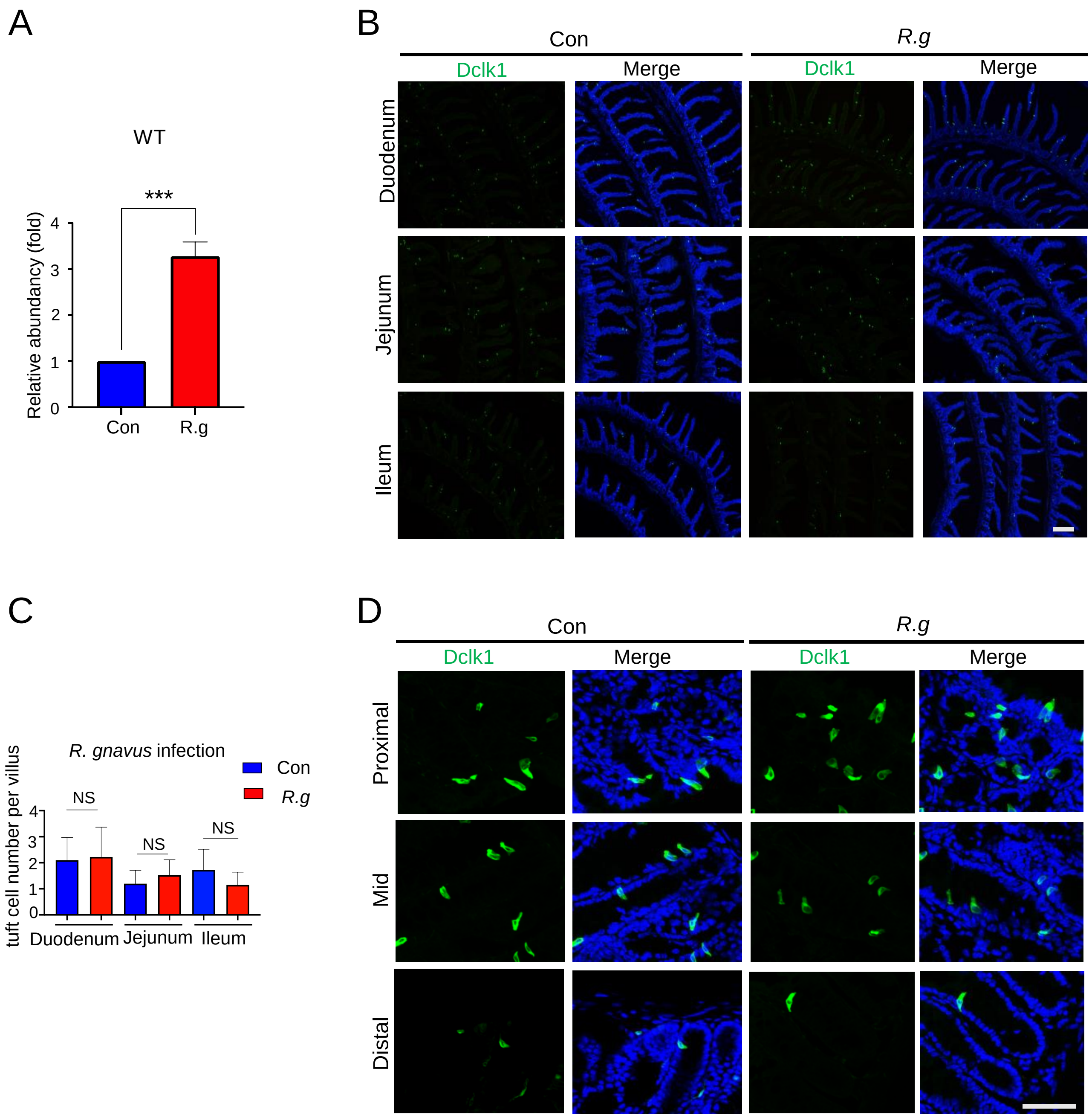

Supplementary Figure 2

R. gnavus infection increases the number of tuft cells in the proximal colon but not in the small intestine. (A) qPCR assays indicate a significant increase of *R. gnavus* in the inoculated fecal samples (R.g) comparing to control samples (Con). Data (means ± SD) were obtained from three repeats, which were tested for normal distribution. Student t tests were performed. ***P<0.001. (B) Immunostaining of duodenal, jejunal and ileal sections with Dclk1 antibody. The number of Dclk1-expressing cells (green) were quantified. Scale bar: 100 µm. (C) No significant difference in the number of tuft cells per villus was found in the duodenum, jejunum and ileum between *R. gnavus*-infected and uninfected wild-type control mice. Data (means ± SD) were obtained from 140 villi of four mice per sample, which were tested for normal distribution. Student t tests were performed. (D) Representative images of anti-Dclk1 antibody staining (green) on the proximal, mid and distal colon of *R. ganvus*-uninfected control (Con) and -infected (R.g) mice, related to Figure 1A, but at a higher magnification. Scale bar: 50 µm.

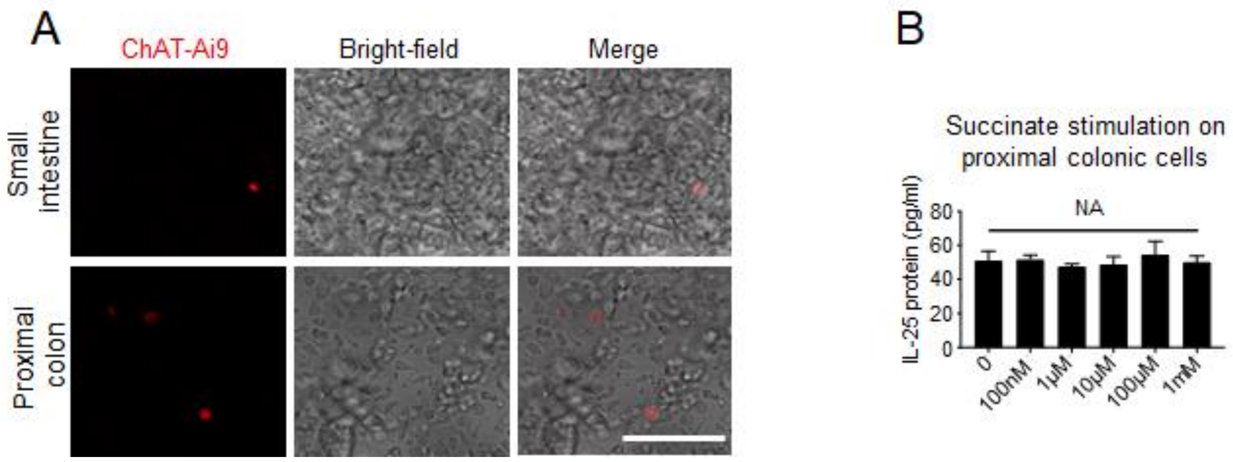

Supplementary Figure 3

IL-25 ELISA assays. (A) Images of the cells isolated from mouse small intestines and proximal colons used for ELISA assays. Note: red fluorescent cells were tuft cells expressing ChAT-Ai9 tdTomato. Scale bar: 100 µm. (B) a series of succinic acid concentrations from 100 nM to 1 mM was unable to evoke IL-25 from the proximal colonic tuft cells. Data (means ± SD) were obtained from three independent experiments, each experiment including three repeats for each data point. The data were tested for normal distribution and One-way ANOVA tests were performed.

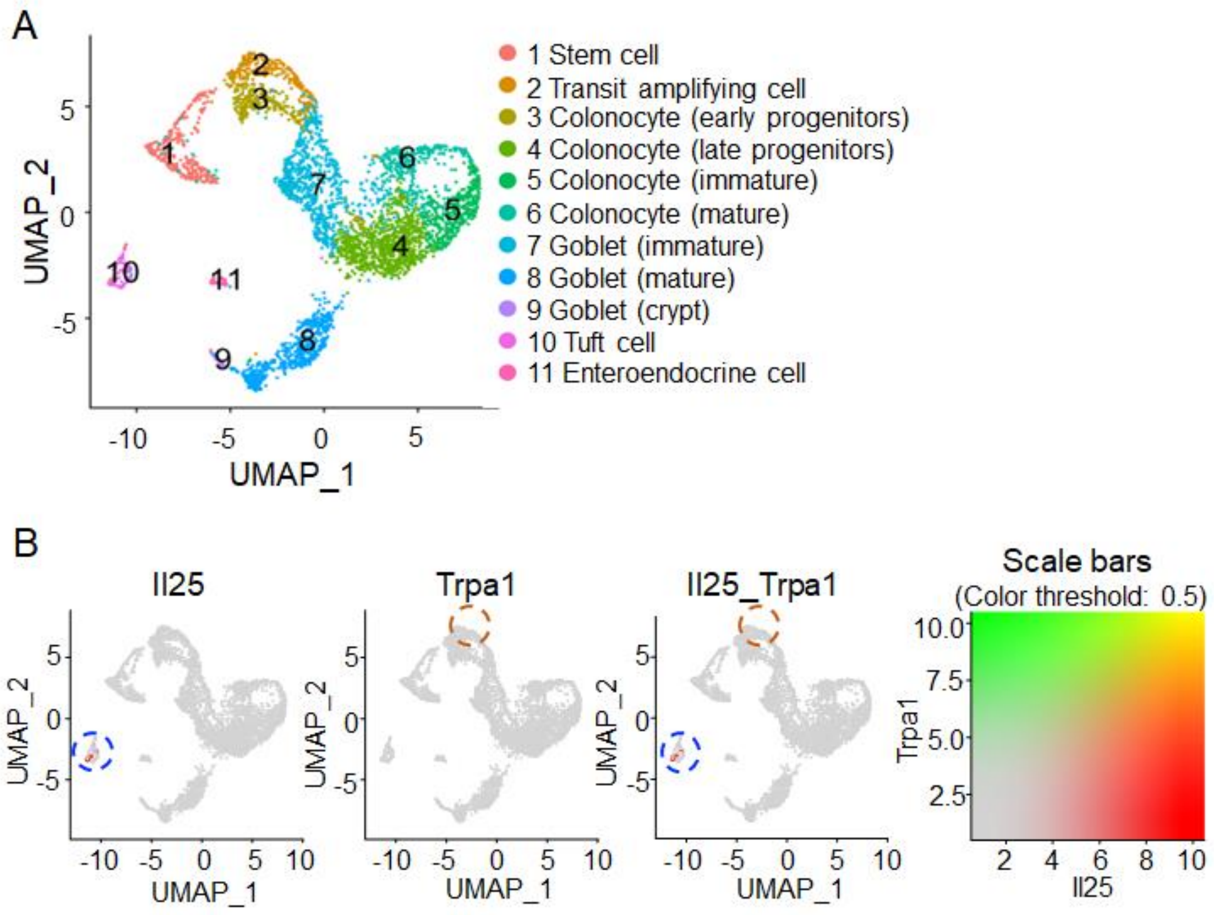

Supplementary Figure 4

The proximal colonic tuft cells do not express TRPA1 ion channel. (A) Uniform manifold approximation and projection (UMAP) analysis shows 11 types of proximal colonic epithelial cells based on single-cell RNAseq data (4). (B) IL-25 was found abundantly only in tuft cells (blue circle) whereas Trpa1 was barely detectable in few transit amplifying cells (orange circle).

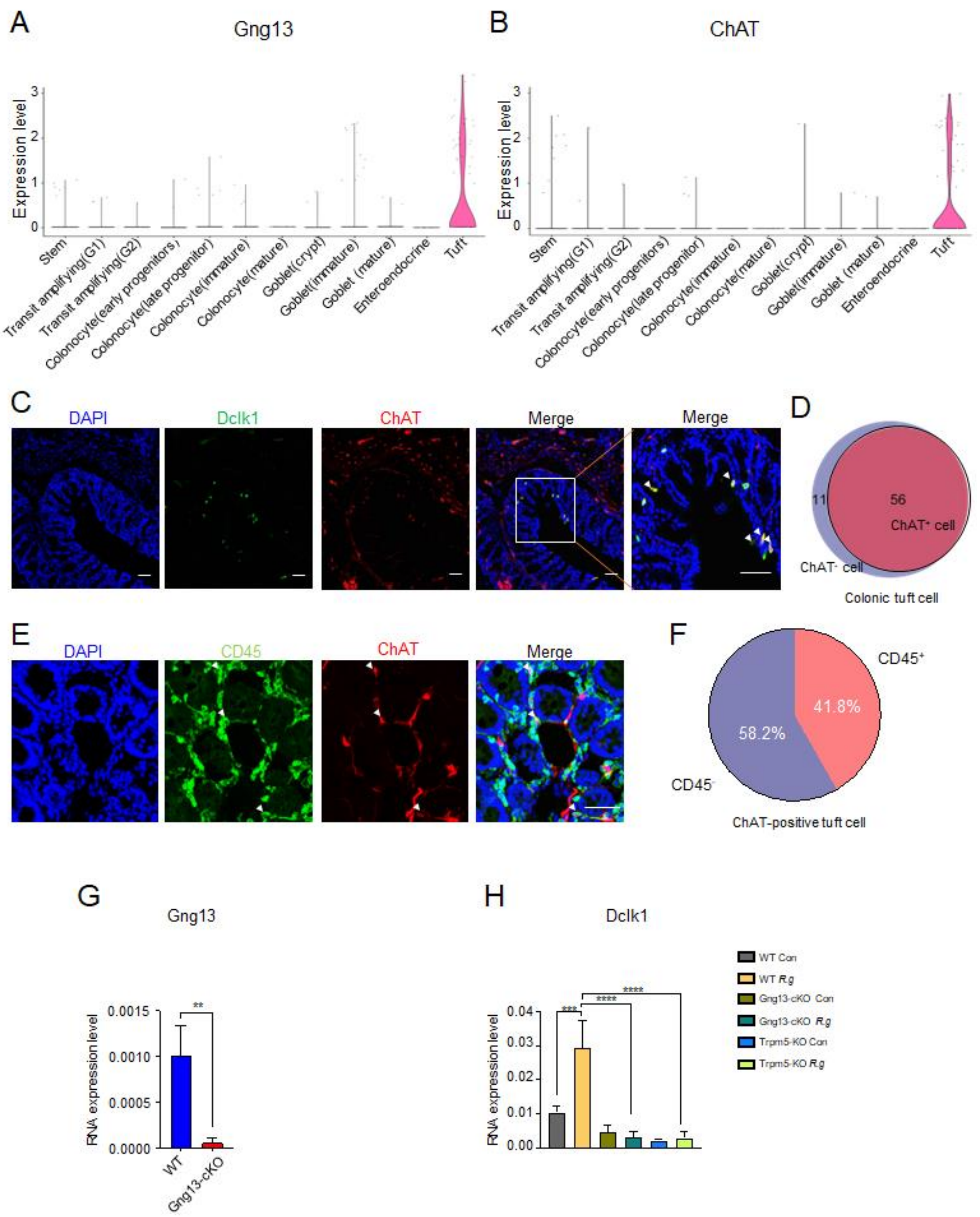

Supplementary Figure 5

Expression of Gng13 and ChAT in the proximal colonic tuft cells. (A, B) Reanalysis of colonic single- cell RNAseq data (3) indicates the co-expression of Gng13 and choline acetyltransferase

(ChAT) in the colonic tuft cells. (C, D) Immunostaining with an anti-Dclk1 antibody on ChAT-Ai9 colonic sections showed that about 83.6% of Dclk1 positive (green) tuft cells express ChAT-Ai9 (red tdTomato). Arrowheads: Dclk1 and Ai9 double positive tuft cells. Scale bar: 100 μm. (E, F) Immunostaining with an anti-CD45 antibody on ChAT-Ai9 colonic sections showed that about 58.2% and 41.86% of Ai9+ tuft cells were CD45- and CD45+ subtypes I and II tuft cells, respectively. Arrowheads: $CD45^+/Ai9^+$ tuft cells. Scale bar: 50 μm. (G) qRT-PCR analysis showed that Gng13 expression in the proximal colon was significantly reduced in the Gng13-cKO mice comparing with WT control. qPCR data (means ± SD) were obtained from three repeats, which were tested for normal distribution. Student t tests were performed. (H) qRT-PCR analysis showed that Dclk1 expression was significantly lower in the Gng13-cKO and Trpm5-KO proximal colons than WT control following *R. gnavus* infection (WT R.g versus Gng13-cKO R.g; WT R.g versus Trpm5-KO R.g). *R.gnavus* (R.g) infection significantly increased Dclk1 expression in WT proximal colon (WT R.g) comparing with the uninfected control (WT con). But the increase did not occur in the Gng13-cKO (Gng13-cKO R.g) or Trpm5-KO (Trpm5-KO R.g) proximal colons following *R. gnavus* infection. qPCR data (means ± SD) were obtained from three replicates and tested for normal distribution. One-way ANOVA tests were performed. **P<0.01; ***P<0.001; ****P<0.0001

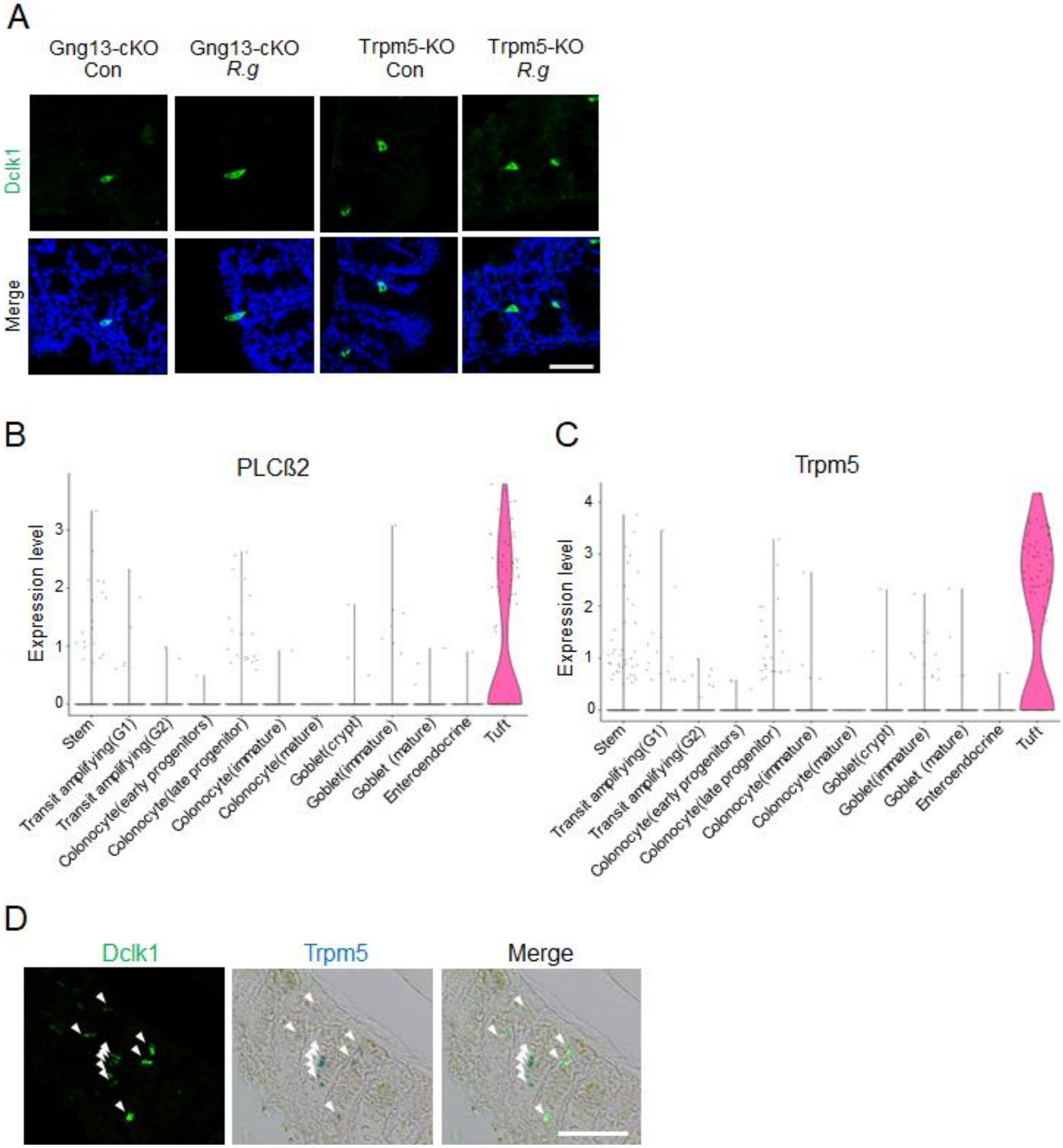

Supplementary Figure 6.

Taste signaling proteins G γ 13, PLCβ2 and Trpm5 are important to the proximal colonic tuft cells' function. (A) Representative images of anti-Dclk1 antibody staining on the proximal colon sections from *R. gnavus*-uninfected (Con) and infected (R.g) Gng13-cKO and Trpm5-KO mice, related to Figure 3A, F, but at a higher magnification. Scale bar: 50 μm. (B, C) Reanalysis of single colonic cell RNAseq data (3) indicates the co-expression of PLCβ2 and Trpm5 in the colonic tuft cells. (C) Representative images of double staining with an anti-Dclk1 antibody (green) and X-gal staining

(blue) on the Trpm5$^{+/-}$ proximal colon sections, confirming colocalization of Dclk1 and Trpm5 to tuft cells. Arrowheads: double stained cells. Scale bar: 100 µm.

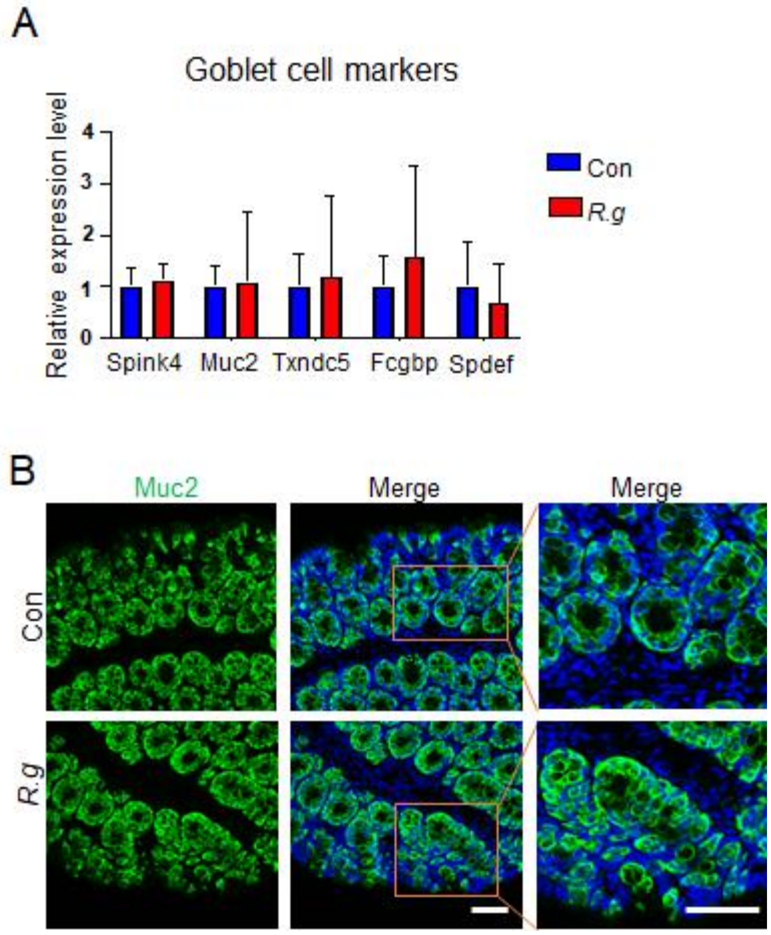

Supplementary Figure 7

qRT-PCR and immunohistochemical analyses of goblet cells. (A) No significant changes were found in the expression of goblet cell marker genes *Spink4*, *Muc2*, *Txndc5*, *Fcgbp* and *Spdef* between *R. gnavus*-uninfected (Con) and -infected (*R.g*) proximal colons. qPCR data (means ± SD) were obtained from three replicates and tested for normal distribution. Student t tests and Mann Whitney tests were performed. (B) Z-stack images of *R. gnavus*-uninfected (Con) and -infected (*R.g*) proximal colonic sections (15 µm thick) stained with an antibody to the goblet cell marker mucin 2 (Muc2), related to Figure 4A but at a higher magnification. Scale bar: 50 µm.

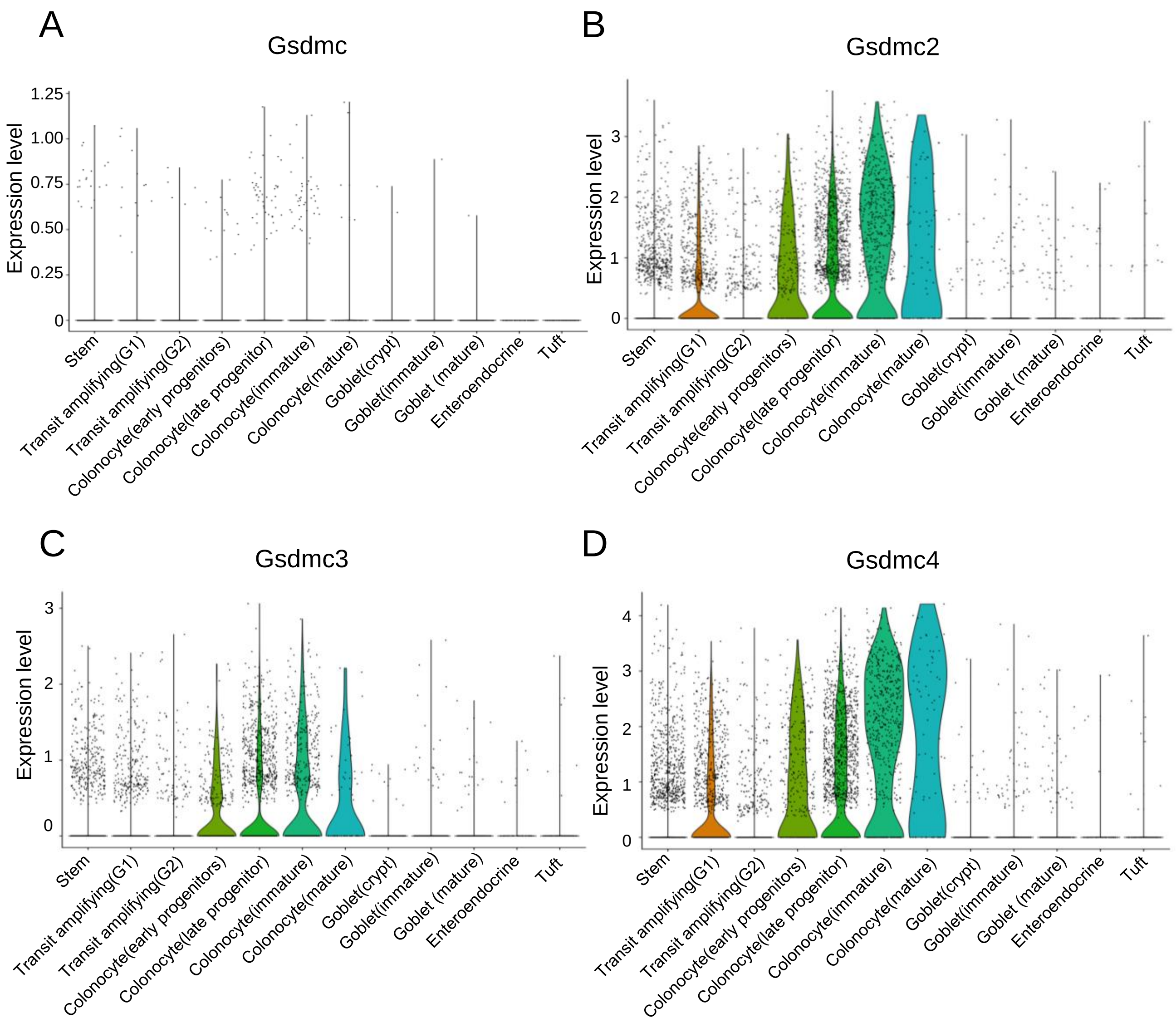

Supplementary Figure 8

Gasdermin C expression in the proximal colonic cells. Reanalysis of colonic single cell RNAseq data indicates that while Gasdermin C (A: Gsdmc) is hardly expressed in the proximal colonic cells, Gasdermin C2, C3 and C4 (B, C, and D: Gadmc2, c3, c4) are abundantly expressed in colonocytes and some stem cells.

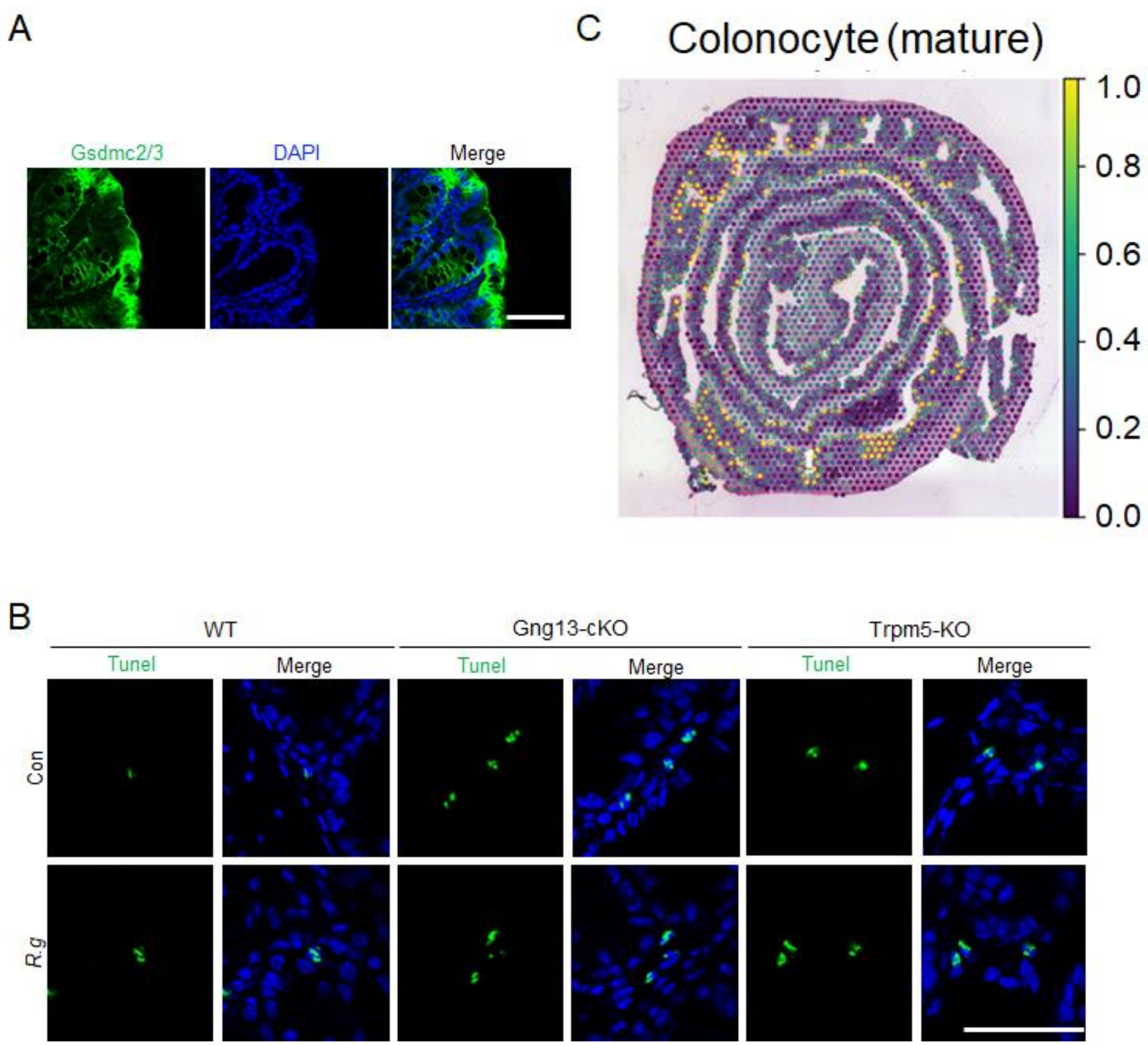

Supplementary Figure 9

Gasdermin expression and TUNEL analysis. (A) Immunostaining of *R. gnavus*-uninfected WT proximal colon sections with an antibody to both Gsdmc2 and Gsdmc3 (Gsdmc2/3), related to Figure 5B but at a higher magnification. (B) TUNEL assays on the *R. gnavus*-uninfected (Con) and -infected (*R.g*) WT, Gng13-cKO and Trpm5-KO proximal colon sections, related to Figure 5D but at a higher magnification. (C) Combined analyses of single-cell RNAseq data with spatial transcriptome data. Single-cell RNAseq data (3) of mouse proximal colonic cells was analyzed along with the spatial transcriptome data (6), identifying mature colonocytes at the top of the proximal colonic crypts (yellow spots). Scale bar: 50 μm.

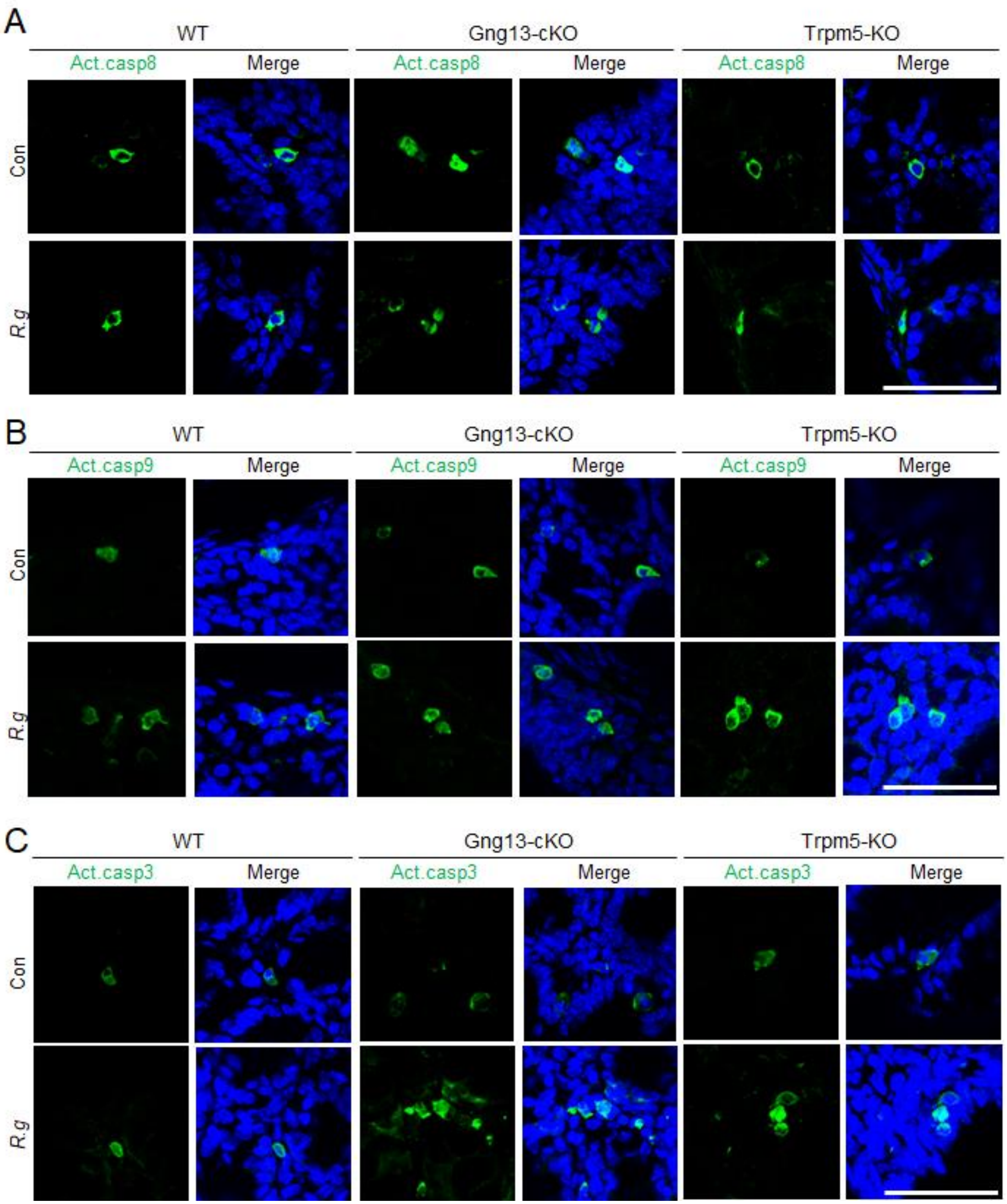

Supplementary Figure 10

Immunostaining of *R. gnavus*-uninfected (Con) and -infected (*R.g*) WT, Gng13-cKO and Trpm5-KO proximal colon sections with antibodies to activated caspases 8, 9 and 3 (A, B, and C), related to Figure 6A, C and E, respectively, but at a higher magnification. Scale bar: 50 μm.

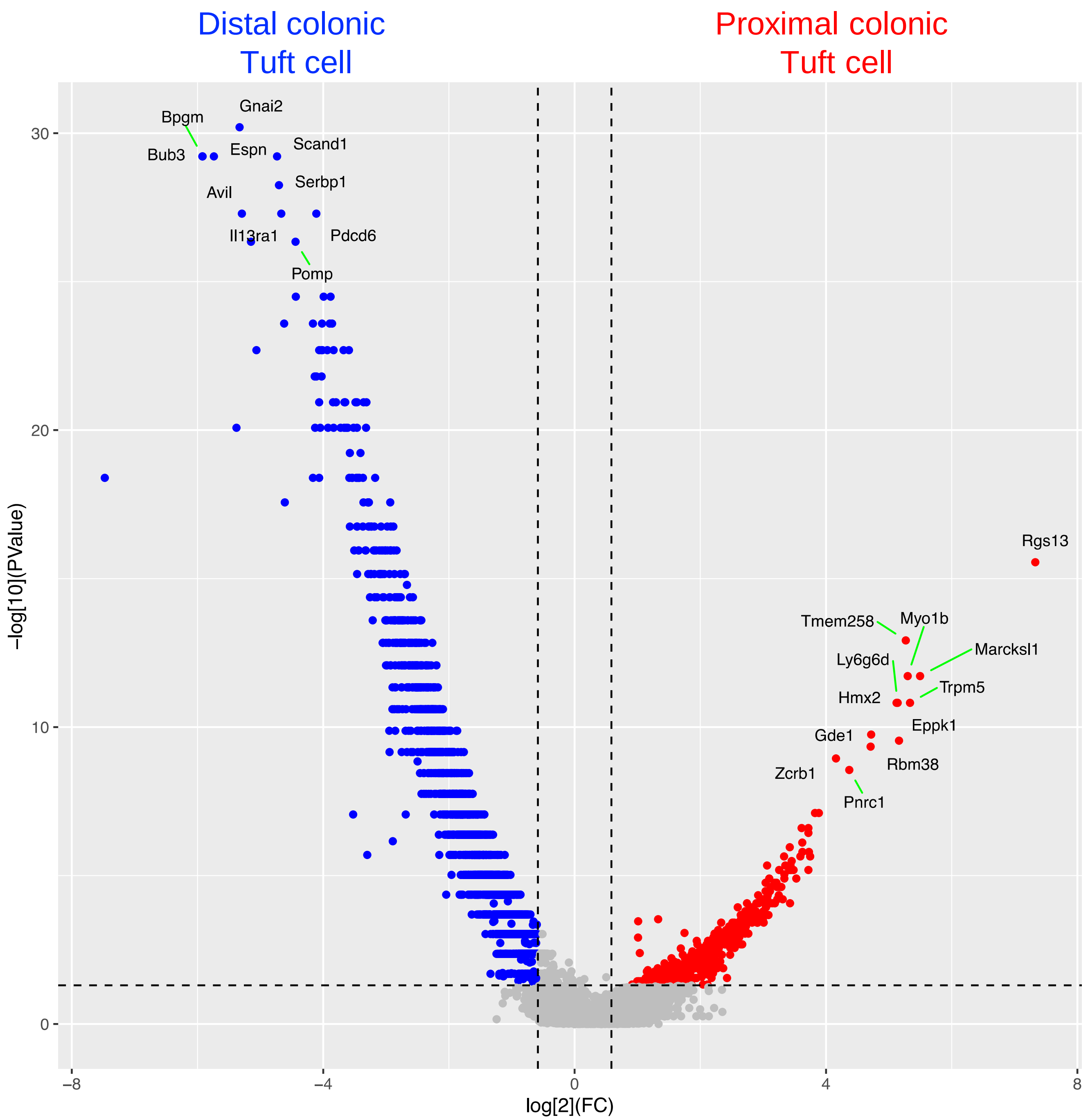

Supplementary Figure 11. Volcano plot of differentially expressed genes between proximal and distal colonic tuft cells based on single cell RNAseq dataset GSE168448 (4). The results indicate that the proximal and distal colonic tuft cells are very different in gene expression profiles. For example, the former highly express *Trpm5* and *Rgs13* while the latter express abundantly *Gnai2*, *Avil1* and *IL13ra1*, suggesting that the two types of tuft cells may have different responsivity to *R. gnavus* and perhaps different physiological functions as well.

Supplementary references:

1. Coletto E, Latousakis D, Pontifex MG, Crost EH, Vaux L, Perez Santamarina E, et al. The Role of the Mucin-Glycan Foraging Ruminococcus Gnavus in the Communication between the Gut and the Brain. *Gut Microbes* (2022) 14(1):2073784. Epub 2022/05/18. doi: 10.1080/19490976.2022.2073784.

2. Wu S, Sun C, Li Y, Wang T, Jia L, Lai S, et al. Gmrepo: A Database of Curated and Consistently Annotated Human Gut Metagenomes. *Nucleic Acids Res* (2020) 48(D1):D545-D53. Epub 2019/09/11. doi: 10.1093/nar/gkz764.

3. Melhem H, Kaya B, Kaymak T, Wuggenig P, Flint E, Roux J, et al. Epithelial Gpr35 Protects from Citrobacter Rodentium Infection by Preserving Goblet Cells and Mucosal Barrier Integrity. *Mucosal Immunol* (2022) 15(3):443-58. Epub 2022/03/11. doi: 10.1038/s41385-022-00494-y.

4. Sirvinskas D, Omrani O, Lu J, Rasa M, Krepelova A, Adam L, et al. Single-Cell Atlas of the Aging Mouse Colon. *iScience* (2022) 25(5):104202. Epub 2022/04/29. doi: 10.1016/j.isci.2022.104202.

5. Hao Y, Hao S, Andersen-Nissen E, Mauck WM, 3rd, Zheng S, Butler A, et al. Integrated Analysis of Multimodal Single-Cell Data. *Cell* (2021) 184(13):3573-87 e29. Epub 2021/06/02. doi: 10.1016/j.cell.2021.04.048.

6. Parigi SM, Larsson L, Das S, Ramirez Flores RO, Frede A, Tripathi KP, et al. The Spatial Transcriptomic Landscape of the Healing Mouse Intestine Following Damage. *Nat Commun* (2022) 13(1):828. Epub 2022/02/13. doi: 10.1038/s41467-022-28497-0.

7. Palla G, Spitzer H, Klein M, Fischer D, Schaar AC, Kuemmerle LB, et al. Squidpy: A Scalable Framework for Spatial Omics Analysis. *Nat Methods* (2022) 19(2):171-8. Epub 2022/02/02. doi: 10.1038/s41592-021-01358-2.

8. Wolf FA, Angerer P, Theis FJ. Scanpy: Large-Scale Single-Cell Gene Expression Data Analysis. *Genome Biol* (2018) 19(1):15. Epub 2018/02/08. doi: 10.1186/s13059-017-1382-0.

9. Biancalani T, Scalia G, Buffoni L, Avasthi R, Lu Z, Sanger A, et al. Deep Learning and Alignment of Spatially Resolved Single-Cell Transcriptomes with Tangram. *Nat Methods* (2021) 18(11):1352-62. Epub 2021/10/30. doi: 10.1038/s41592-021-01264-7.

10. Yeoh YK, Zuo T, Lui GCY, Zhang F, Liu Q, Li AYL, et al. Gut Microbiota Composition Reflects Disease Severity and Dysfunctional Immune Responses in Patients with Covid-19. *Gut* (2021) 70(4):698-706. doi: 10.1136/gutjnl-2020-323020.

Supplementary Table 1

sequences of qRT-PCR primers		
Gene	Forward Primer	Reverse Primer
mTas2r102	CTCCTGCTAATCTTCTCTTTGTG	GGGTCTCTGTGTCTTCTGG
mTas2r103	AGCACAGTGGCCCACATAAA	TGGCCTGTGGGAAAAGCTAC
mTas2r104	GCAACACATCCTGGCTGAT	CCCCATATTGGCAAAAACAT
mTas2r105	AAGGCATCCTCCTTTCCATT	GTGCAATAAATGTGTTCCCTAAAA
mTas2r106	AGCCACATTCTTCTCAACCT	AGCATGTAATGATAGCCACCA
mTas2r107	GGCATCCTCCTTTGTGTTGT	TGCAATATATGTGTCCCCTAAAAC
mTas2r108	GTTTCTCCTGTTGAAACGGACT	GTGAGGGCTGAAATCAGAAGA
mTas2r109	GTCAAATTCAGGTGTTAGGAAGTC	CACAGGGAGAAGATGAGCAG
mTas2r110	CTTTCTCATGCTCATCTTCTCAC	GGCATCTCTAGGTGGTTTGG
mTas2r113	CCACGGTAATGTTTTCTTTGC	TGGTGCTGATGTCTCTGCAT
mTas2r114	CGGCTGCCACTCACTTATC	CAGCACTTTAATAGTTGCAGTATCATT
mTas2r115	CCTTTGGTGTATCCTTGATAGCTT	CTGCATCTTCCTTACATGTTTCA
mTas2r116	AAGGTTTGGAGTGCTCTGCT	AGCTGTTCTTGCAACCTGTGT

mTas2r117	CCCTGTGGACACATCACAAG	TCACAGTTTGTAGGGCTTTGAA
mTas2r118	CACTGGGTGCAGATGAAACA	CTTCAGAACAGTGAACTGAGCTTT
mTas2r119	AAGGAACCCAAGACTCAGTGAC	AGGCTTCTGAGCAGGATGTC
mTas2r120	TGTTAACGAACTGGCATTCAC	GGTTGGTTATAGCCCAGGT
mTas2r121	CTGGTCTTATTGGAGATGATTGTG	GGAGAAGATTAACAGGATGAAGGA
mTas2r122	TCTTCTCTTTATGGAGCCACCT	GCTTCTGTGCTTATGTCTTTGG
mTas2r123	CATTAAAGCCTTGCAAACTGTG	GGAAAAGTAAGTATATGGCATACAGCA
mTas2r124	CTACGGCCCACAGAAATGCC	AGCTGCCTCATTACCCAAAGA
mTas2r125	AAGGCCTTGCACATGGTAGT	GGCAAGAGACAAAAAGAAAACTG
mTas2r126	GTGTGTGGGATTGGTCAACA	GCTCCCGGAGTACTCAACC
mTas2r129	TTTAGCATGTGGCTTGCTGC	AGAGGCCCAAAGACATGAGC
mTas2r130	TGCATTCATTGCACTGGTAAA	GATTAAATCAATAGAGGCAATCTTCC
mTas2r131	TAGCCCACATTTCCCATCC	CAAGCACACCTCTCAATCTCC
mTas2r134	GCCTGGGAAGTGGTAACCTA	GTTGCTTAGTATCAGAATGGTGGA
mTas2r135	CCATCATGTCCACAGGAGAA	TCAGTAGTCTGACATCCAAGAACTGT

mTas2r136	GGACAATGAGGCTTTATGGAA	CCTTAATGTGGGTTGAAGCAC
mTas2r137	CTGGCTCAAATGGAGAGCTT	GGTACTGACACAGGATAAGAGCAG
mTas2r138	CAAACCAAGTGAGCCTCTGG	GAGAAGCGGACAATCTTGGA
mTas2r139	ATGGCTCAACCCAGCAACTAC	ACAGCCATGACAATCCCACT
mTas2r140	GAAGAACATGCAACACAATGC	AGGGCCTTAATATGGGCTGT
mTas2r143	CATTGGCCTCTATGTTGCAG	TGTCCGGTTCCTCATCCA
mTas2r144	AAGCAGAAAATCATAGGGCTGA	TGAAGGAAACCAACACTGACA
mouse GAPDH	CGACTTCAACAGCAACTCCCACTCTTCC	TGGGTGGTCCAGGGTTTCTTACTCCTT
mDclk1	TGAACAAGAAGACGGCTCACTCC	GCTGGTGGGTGATGGACTTGG
mPlcß2	CCTGGAGGTGACAGCTTATGA	GCTCCGTGAAGGAAGAGACA
mTrpm5	GCAAATCCCTCTGGATGAAA	TAGCTGAACATGGCGATCAG
mSucnr1	ACAGAAGCCGACAGCAGAAT	GCACAGGAAAGCAAAGTCAG
mGnat3	CTGAGCGGGATGCAAGAACT	CTTGGTCCTCTCGGCTCCTA
mGng13	ATGGAGGAGTGGGATGTGC	GCTCGGGGATGGTCTTGGACG
mGsdmc	TCTTCCCGGTTGGCTTTGAAA	AGGACTTAACAAACCCTGCTTC
mGsdmc2	CTGTGGAATGCTTGTCCGATG	CCTCCAGGTCCGTTGATTGG
mGsdmc3	AGCCCGCCCATCTAGATTTC	TGCCCCAACTGACTCAACTC

mGsdmc4	TGAGGAGCCTGCCAATCTAAA	ATGTGGGGTGCTAGAATCCTT
Rg 16s 5	TGGCGGCGTGCTTAACA	TCCGAAGAAATCCGTCAAGGT
mAim2	ACAGCAGGTTTAAACACACGAG	AGCACGACTTCTAGCCCCTA
mCasp1	AGGCACGGGACCTATGTGAT	AGGGCAAAACTTGAGGGTCC
mCasp3	TGGCTTGCCAGAAGATACCG	CCGTTGCCACCTTCCTGTTA
mCasp4	TGTCTTCACGGTGCGAAAGA	CCTTTCGTGTACGGCCATTG
mCasp6	AAGTGTTCGATCCAGCCGAG	CAGGTTGTCTCTGTCTGCGT
mCasp8	CCTCCGAGAGTGCTGACATT	ACTAGGGCATAGGGGACAGA
mCasp9	CTTAGCCCGGAATCCTGCTT	TGCCATTCTGAACTGTGAGGG
mElane	CTTCATCCGAGGAGGCTGTG	GAGGTCTCTGGTAGAGGGGG
mGpx4	GGTTTCGTGTGCATCGTCAC	GGGCATCGTCCCCATTTACA
mGsdma	GAGGTAGGTGCACGGCTTAC	TTCGCCCCACTGGACTTAAC
mGsdma2	TGGAGGCTAGCTGATGTGTC	GCGTTTGAAGTCGATGAGGC
mGsdmd	TCGGGCTGAAGCTTTACGG	CTGCCGCTTACCTCCTTGAT
mIl18	CAGTGAACCCCAGACCAGAC	ACAGGCGAGGTCATCACAAG
mIl1ß	TGTCTGAAGCAGCTATGGCAA	TAGCCCTCCATTCCTGAAAGC
mIl6	GCCCACCAAGAACGATAGTCA	TTGTGAAGTAGGGAAGGCCG
mNlrc4	AGAATCGCTATGCTCTGGGC	AAGGTCGGCTTGCTGATGAA
mNlrp1a	AAGAAGCTCTCACCCTGACTC	TTTCAGGAAGGCAGGCATCAG
mNlrp1b	TTGTGGCTTGTTGAATGCGG	AGCTGCAGGTCTAGCTCTCT
mNlrp2	AAGGTCCCCGGATGAACAAC	TCCAGTGCAGAGCTGTTGAG

mNlrp3	TATCCACTGCCGAGAGGTGA	TCTTGCACACTGGTGGGTTT
mNlrp6	GGGGTGCTTTGGAGGTTCA	CCCTTAGCTTGTGGCGGAA
mNod1	TGACGTTCCTGGGACTAGGG	TTGCCCCTTCGTCTCCAATC
mNod2	GGACGAGTTCAAGTTCCGGT	GGCATCTGGACACCATCCAA
mPlcg1	TCTGGCGGAATGGGAAAGTC	TTCGTCTGTGGAACAGGCTC
mPrkaca	CAACTTCCCGTTCCTGGTCA	AGGTCCCGGTAGATGAGGTC
mPycard	GTCTTAGGGGCGGAAACCAA	CCGCGGTCACCTTTTACTCT
mScaf1	TGCAGAGCTATTACTCGCGG	ACAGGCAGCTGAGAACCATC
mTirap	ACAATGGAGACACCAAGACC	GGAGGGCATTTGAGATCCGT
mTnf	CGGGCAGGTCTACTTTGGAG	ACCCTGAGCCATAATCCCCT
mTph1	GCGCCCGTTTGGACTGAAGTA	GCCCTGGCTCTAGACTGATGCTC
mChga	AGAACCAGAGCCCTGATGCCAA	CTCTGTGGTTGCCTCAAAGCCA
mChgb	TCTACGACTCGGAGGAGCAGAT	CCGCTATCTTCTGGAGTTCCAG
mMuc2	CTGACCAAGAGCGAACACAA	CATGACTGGAAGCAACTGGA
mFcgbp	CCATCCACAAGAATGAGATCGGC	GTCAGTCTCCAGCACAGCCTTT
mTxndc5	TGTCACCATCGCAGAAGTGGAC	GAGGTCTCTACCTCCGTTGTGT
mSpdef	ACGTTGGATGAGCACTCG	CCATAAAAGCCACTTCTGCAC
mSpink4	TATGCATCTGTGGCTGGTCA	GCTCAGCCATGTGCTCACAG
